# Implicit and explicit: a scoping review exploring the contribution of anthropological practice in implementation science

**DOI:** 10.1186/s13012-024-01344-0

**Published:** 2024-02-12

**Authors:** Elissa Z. Faro, Peter Taber, Aaron T. Seaman, Ellen B. Rubinstein, Gemmae M. Fix, Heather Healy, Heather Schacht Reisinger

**Affiliations:** 1 Department of Internal Medicine, Carver College of Medicine, University of Iowa, Iowa City, IA USA; 2 Department of Biomedical Informatics, University of Utah School of Medicine, Salt Lake City, UT USA; 3 Center for Access and Delivery Research and Evaluation (CADRE), Iowa City VA Health Care System, Iowa City, IA USA; 4 Department of Sociology and Anthropology, North Dakota State University, Fargo, ND USA; 5 Center for Healthcare Organization and Implementation Research (CHOIR), VA Bedford and VA Boston Healthcare System, Bedford, MA USA; 6 Boston University Chobanian & Avedisian School of Medicine, Boston, MA USA; 7 Hardin Library for the Health Sciences, University of Iowa, Iowa City, IA USA; 8 Institute for Clinical and Translational Science, University of Iowa, Iowa City, IA USA

**Keywords:** Ethnography, Implementation science, Scoping review, Anthropology, Qualitative, Social science

## Abstract

**Background:**

This study’s goal is to identify the existing variation in how, why, and by whom anthropological practice is conducted as part of implementation science projects. As doctorally trained anthropologists, we sought to characterize how and why the term “ethnography” was variously applied in the implementation science literature and characterize the practice of anthropology within and across the field.

**Methods:**

While we follow the PRISMA-ScR checklist, we present the work with a narrative approach to accurately reflect our review process. A health services librarian developed a search strategy using subject headings and keywords for the following databases: PubMed, Embase (Elsevier), Cochrane CENTRAL (Wiley), CIHAHL (EBSCO), PsycINFO (EBSCO), Web of Science Core Collection, and Anthropology Plus (EBSCO). We focused on the practice of anthropology in implementation research conducted in a healthcare setting, in English, with no date restrictions. Studies were included if they applied one or several elements of anthropological methods in terms of study design, data collection, and/or analysis.

**Results:**

The database searches produced 3450 results combined after duplicates were removed, which were added to Rayyan for two rounds of screening by title and abstract. A total of 487 articles were included in the full-text screening. Of these, 227 were included and received data extraction that we recorded and analyzed with descriptive statistics in three main domains: (1) anthropological methods; (2) implementation science methods; and (3) study context. We found the use of characteristic tools of anthropology like ethnography and field notes are usually not systematically described but often mentioned. Further, we found that research design decisions and compromises (e.g., length of time in the field, logistics of stakeholder involvement, reconciling diverse firsthand experiences) that often impact anthropological approaches are not systematically described.

**Conclusions:**

Anthropological work often supports larger, mixed-methods implementation projects without being thoroughly reported. Context is essential to anthropological practice and implicitly fundamental to implementation research, yet the goals of anthropology and how its practice informs larger research projects are often not explicitly stated.

**Supplementary Information:**

The online version contains supplementary material available at 10.1186/s13012-024-01344-0.

Contributions to the literature

- There is a tension between the epistemology of ethnography as developed and used by anthropologists versus the suite of qualitative methods employed by implementation science.
- Regular use of characteristic tools of anthropological methods like observation and field notes are usually not systematically described.
- Context is essential to anthropological practice and implicitly fundamental to implementation research, yet the goals of anthropology and how its practice informs larger research projects are often not explicitly stated.
- Researchers trained in diverse traditions contributing to implementation research can make explicit their theoretical and methodological contributions to enhance rigor and reproducibility.

## Background

As implementation science has become an established, methodologically rigorous, and theoretically informed field in its own right, there has been increasing interest in unpacking what goes into good implementation science [1, 2]. Since its inception, implementation science has been an inherently interdisciplinary field, drawing theoretical and methodological approaches from many social sciences, including psychology, sociology, anthropology, economics, and organizational studies [3]. Further, implementation science has been adopted, adapted, and deployed in many different contexts, such as *knowledge translation* in Canada. However, there is often little cross-communication across these contexts. Even within the USA, implementation scientists may not be aware of colleagues in different institutional contexts. Whenever a field draws together so many disciplines and approaches, each with its own rich history, its practitioners benefit from ongoing dialogue about cross-disciplinary theoretical and methodological adoptions and adaptations.

As a group of six doctorally trained four-field anthropologists working in implementation and adjacent fields, we sought to characterize the use of anthropological approaches and methods in implementation science. This review encompassed not only the use of data collection and analytic methods traditionally associated with anthropology—such as ethnography, iteration, and triangulation—but also by whom and how these methods are practiced and described in the implementation science literature.

There has been a recent increase in research that seeks to characterize how implementation science uses methods from different fields across different contexts. Subsequently, we take as our starting point work by Gertner and colleagues and Hagaman and colleagues respectively reviewing the use of ethnographic approaches specifically and qualitative methods more broadly in implementation research [4, 5]. Gertner et al.’s scoping review results and Hagaman et al.’s scoping review protocol and presentation of preliminary results provide important, foundational work on the use of qualitative and ethnographic methods and terminology that ground our current scoping review [6]. Their respective work enabled our team of co-authors to investigate the more implicit and less well-defined uses of anthropological methods in implementation science. We were able to hone our analysis to explore the relative invisibility of anthropologists doing work in implementation science and the reasons why and how this invisibility occurs.

There are challenges inherent in the adoption and adaptation of the methods of one field into another, as here with anthropology in implementation science. One particularly salient example, and the major crux of our scoping review, is ethnography. Although ethnographic methods are used widely by sociology, nursing, psychology, and other social sciences, anthropology’s fundamental means of understanding and knowledge production is the “socially embedded realism of participant observation” that is distinct from the deployment of ethnography by other disciplines [7]. As demonstrated by the recent reviews by Gertner et al. and Hagaman et al., tension exists between ‘ethnography’ in implementation research—as one method in the data collection toolkit (e.g., a combination of observation and interviews)—and ethnography as a fully realized theoretical and methodological approach that comprises its own meaningful epistemology [8–10]. For example, nursing research considers ethnography as one of the three styles of qualitative research, along with phenomenology and grounded theory [11].

While other disciplines have adopted the qualitative components of ethnography into their own constellation of methods, in a recent reflection on ethnographic thinking in a special issue celebrating 50 years of *American Ethnologist*, Emanuel Moss points out that “method without theory is insufficient” [12]. In the same issue, other authors comment on anthropology’s approach to learning *from* people (rather than about them), and anthropological fieldwork comprising being *with* rather than simply being there [7, 13]. Anthropologists’ practice of ethnography lacks a clear-cut definition not only because it is an amorphous but distinct combination of epistemology, theory, and methods but also because anthropological ethnography is read by other anthropologists, negating the need to be explicit [14]. This disciplinary tension underpins our exploration of ethnography within implementation science, in order to disentangle descriptions of qualitative methods from anthropological praxis.

In this scoping review, we sought to characterize how the implementation science literature describes anthropological practice broadly, including the explicit use of methods such as ethnography in the Gertner review; in comparison and contrast to the suite of qualitative methodological approaches in the Hagaman review; and in the more implicit, epistemological approaches to understanding how people see the world and make sense of their actions in it. This endeavor demanded an operationalization of ethnography in much the way that ethnography as a practice forces practitioners to have more explicit conceptualizations of both familiar and unfamiliar social and cultural forms in the domain of interest (e.g., “the family”, “economy”, “environment”, “health”) [15]—that is, to make the strange familiar and the familiar strange to better understand them both [16].

Broadly, anthropological practice is “the total context whereby the researcher acquires knowledge through experience” ([17]; p. 5). Our many iterations of developing and then rejecting definitions of ethnography through the process of this scoping review reflected our discomfort both with methodological gatekeeping but also with the positivist demands for generalizability and reproducibility. By default, we adopted a hybrid approach where we had a priori questions about how anthropological practice is described in the implementation science literature but let our iterative process of close reading, discussion, and interpretation guide the process of the review. We, ultimately, were seeking articles that reflected anthropological practice but did not fully realize this objective until that iterative process of reading the final 227 articles informed our thinking.

## Methods

We chose a scoping review framework because the exploratory, iterative nature of this method best fits our goals of characterizing the broadest possible extent of an anthropological and ethnographic epistemology in implementation science, without the need to assess the quality of the studies. We conducted our review using the framework outlined by Arksey 2005 and expanded by Peters 2015 [18, 19]. We also acknowledge our positionality as anthropologists who brought a more ethnographic sensibility to the review process [20]. More specifically, we recognize that the process of reviewing published research to objectively evaluate it as “good/included” was intentionally problematized in the self-reflexive way we conduct all anthropological research to make the familiar strange and to critically examine our assumptions about the world around us [16]. We were hesitant to operationalize ethnography; our process and the discussions about it informed our analysis in real time; and the approach we finally adopted was to characterize the theoretical and methodological approaches that reflected an anthropological orientation using experience and fieldwork to understand how people conceptualize the world around them. We never decisively operationalized a definition of ethnography; we used multiple iterations of reading and discussion by at least two co-authors to determine if the manuscript comprised a description of anthropological practice. To ensure best practice for transparency in reporting our scoping review methodology, we have followed the PRISMA-ScR checklist, as outlined by Tricco 2018 [21], but in keeping with an anthropological approach, we have adopted the following description of the methodology to more accurately represent the iterative, narrative process of our review.

### Data sources and searches

**Fig. 1:**
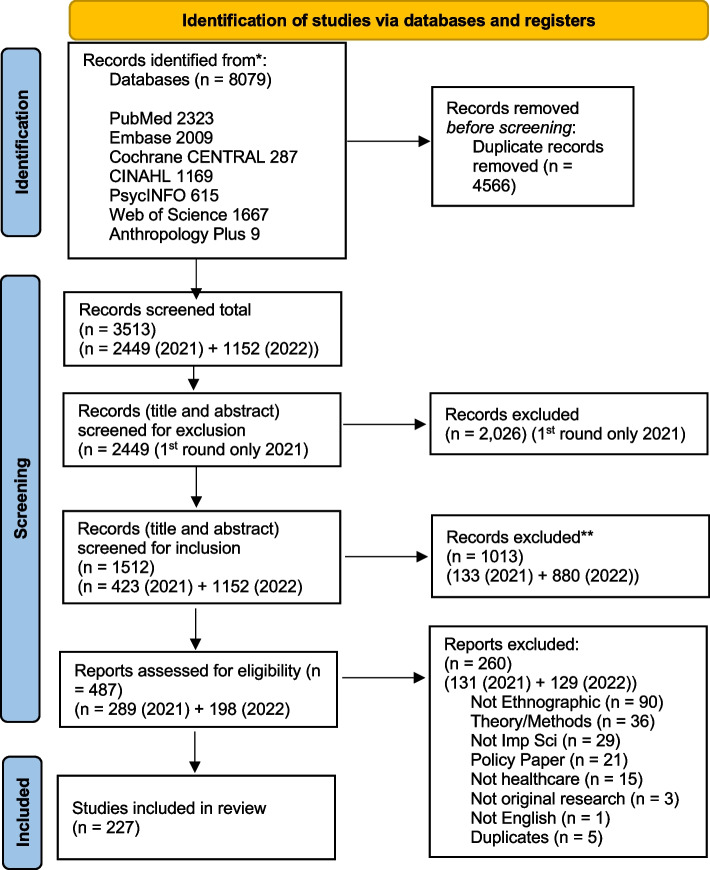
PRISMA 2020 flow diagram for new systematic reviews which included searches of databases and registers [30]

The searches were developed and conducted by a health sciences librarian trained in evidence synthesis searching. The librarian developed a search strategy using subject headings and keywords for the following databases: PubMed, Embase (Elsevier), Cochrane CENTRAL (Wiley), CIHAHL (EBSCO), PsycINFO (EBSCO), Web of Science Core Collection, and Anthropology Plus (EBSCO). The strategies were peer-reviewed by another health sciences librarian trained in evidence synthesis searching. The searches were run on February 15, 2021, with no date or language limits applied. A search update was conducted on September 12, 2022. The strategy for each database is available in Supplemental file 1. All database results were exported to EndNote, duplicates were removed using a multi-step process, and results were transferred from EndNote to Rayyan for screening. The number of records retrieved for each database can be found in Fig. 1.

### Eligibility criteria

This review focuses broadly on implementation science in health-related settings as is it conducted globally, so we considered studies that include healthcare in any country or health system, at all levels of the healthcare system. All English-language articles published in scholarly journals where the full text was available were included. The contents of the manuscripts were focused on the practice of anthropological methods in the context of implementation research conducted in a healthcare setting, including inpatient and outpatient (primary and specialty) care, emergency department, long-term care, and rehabilitation and community health facilities. We included publications that reported on research about some stage of implementation (from pre-implementation through sustainment and de-implementation) of evidence-based practice or intervention focused on improving health. Studies were included if they applied one or several methodological elements of anthropological practice in terms of study design, data collection, and/or analysis, including if “ethnograph*” or “anthropolog*” was specifically mentioned. In lieu of an operational definition of ethnography, we also included articles that described some combination of theoretical/methodological approaches that reflected a deep engagement with people to understand their sensemaking of and in their own context. The words that signaled this approach included but were not limited to longitudinal, participatory, iterative, reflexive, comparative, and in situ. We also looked for research that used multiple data collection methods including focus group discussions, interviews, field notes, observation, site visits, surveys, and document review in combination with theoretically informed analytic approaches including constant comparison, triangulation, and immersion crystallization.

Citations focusing on the practice of anthropology in settings other than healthcare (for example, educational settings) were excluded. In addition to context, we excluded citations by publication type (i.e., review, protocol, methods, policy, abstract, dissertation), data collection methods (e.g., single method only—interview, focus group, survey), and if we determined it was not implementation science (e.g., not the study of uptake of a health-focused intervention). Given the broad range of qualitative methods used in implementation science, we reviewed all studies that employ these methods (e.g., stakeholder, in-depth, semi-structured interviews) but ultimately rejected some based on a lack of the combination of study design, theoretical basis, data collection and/or analytic methods that comprise anthropological practice.

### Article screening and data extraction

We conducted several rounds of reviews. In the first round of abstract reviews conducted in winter 2021, all six research team members conducted the title and abstract screening process. We began with a calibration exercise with a sample of 50 articles prior to the full title and abstract screening. After discussion, each title and abstract record was screened for exclusion by two reviewers, with a third reviewer adjudicating any disagreements. Initial exclusion criteria were based on preliminary definitions of ethnography and implementation science to explore the applicability and general agreement among reviewers, as well as language, study type, if it described original research, or whether the intervention was health-focused. This initial screen gave us a better sense of the landscape, especially for those of us less familiar with the implementation science literature. We then conducted a second round of title and abstract screening for inclusion using the same process of two reviewers and a third adjudicator. In this round, we felt we had an interstitial understanding of the breadth of the descriptions of anthropological practice from the initial exclusion round, so we developed an abstract screening tool (see Supplemental file 2, Abstract Screening Tool).

Developing inclusion criteria was time-consuming and required the most discussion; our group had very different opinions about how traditionally we should define the practice of anthropology and how closely connected to ethnography that practice is, based in part on our own research experiences, professional roles, and affiliations, and how we discussed our own work, both at the front end of the research endeavor (i.e., grant proposals) all the way through to final products (i.e., conference presentations, peer-reviewed publications). Finally, all six research team members conducted the full-text screening, with two reviewers for each article. Discrepancies were discussed and resolved collaboratively. All six members of the research team piloted the data extraction worksheet used for the full-text review with eight articles, and then we iteratively revised it in conversations based on the pilot review results. We collected data on the article characteristics, details on the data collection and analysis methods, the health intervention focus and context, and details of implementation science theories and methods. These data were entered into a spreadsheet for synthesis and reporting.

In fall 2022, a search update was run with the same search terms. The six research team members conducted another title and abstract screening, in which one reviewer screened each title and abstract using the abstract screening tool. All six research team members conducted full-text screening, with one reviewer for each article, using a data collection worksheet that was streamlined based on the prior round of full-text data abstraction. These data were entered into a spreadsheet and combined with the results from the initial round of data extraction for updated synthesis and reporting.

### Reflexive approach to full-text analysis

The development of our data extraction worksheet was an iterative process based on our multiple rounds of piloting, review, and discussion. We were hesitant to decide a priori what would be important to capture and how to standardize what we recorded from each manuscript because, as anthropologists, our realist, iterative approach meant the more abstracts, and then full-text articles, we read, the more our opinions changed about what the field looked like, and what should be included or excluded. The approach we finally adopted was to describe what was out there, with some very broad boundaries, in terms as close to how the included manuscripts presented their own work as possible. Ultimately, we use an ethnographic approach to learn from the research itself, which resulted in less strict definitions and, ultimately, less reproducibility. Even with a standardized data collection spreadsheet, there was a great deal of heterogeneity in how we captured data (i.e., level of detail on open-text fields) given each research team member’s views on what was necessary and important to include.

### Data synthesis and analysis

In the spring and fall of 2022, the research team presented the initial round of search results at two conferences for peer feedback from applied anthropologists and implementation scientists. At the Society for Applied Anthropology Annual Meeting in spring 2022, our full research team presented our progress in a panel discussion that raised important questions and generated fruitful discussion from anthropology peers. That same fall, one team member (EZF) presented the initial results of the data extraction in a poster at the Society for Implementation Research Conference to implementation research peers, which generated different and equally important questions. Initial descriptive statistics were performed with the results initially by two research team members (EZF and PT) that were included in the presentations in 2022.

In the spring of 2023, once the final data extraction had been completed and the results cleaned, additional statistical analyses were performed looking at co-occurrence of data collection and analysis methods as well as other patterns in the dataset. Additional bibliometric and networking analyses were conducted with colleagues in library sciences using Scopus, SciVal, and VOSViewer [22, 23]. SciVal is a tool that reports research performance metrics, based on the Scopus database, which incorporates more than 32 million publication records from almost 22,000 journals from 5000 publishers across the globe [22]. VOSViewer is software for clustering analysis, which we used to identify the network relationships between authors included in our dataset [23]. Scopus and VOSViewer allowed us to identify networks of collaborations and other bibliometric trends over time.

## Results

The database searches produced 3450 results combined after duplicates were removed, which were added to Rayyan for two rounds of screening by title and abstract. A total of 487 articles were included in the full-text screening. Of these, 227 were included as describing anthropological practice in implementation science and received data extraction, which we recorded and analyzed with descriptive statistics. See the PRISMA workflow diagram (Fig. 1) for the complete review process, with details from the search, review, and selection processes included. Because the screening process was updated between the first and second searches, we have included details with dates (first search in 2021, and second in 2022) to clarify the process.

### Study characteristics

Of the 227 included articles (Supplemental file 3), we recorded and analyzed with descriptive statistics three main domains: (1) the use of data collection and analysis methods; (2) implementation science methods; and (3) study context (Table 1). The table included as Supplemental file 4 includes additional selected attributes of the included articles. The three categories of data abstracted together with our bibliometric analyses allowed us to answer our primary questions about who is conducting implementation research with anthropological approaches, how they are describing what they have done, and where and with whom they are doing this work.

**Table 1:** Characteristics of included articles

Characteristic	Number of studies
Anthro/ethno methods	
Use of term “ethnography”	73
Use of term “anthropology”	18
Interrater reliability	11
Data collection	
Observation	118
Field notes	134
Site visits	127
Focus groups	65
Interviews	198
Document review	96
Survey	65
Other	181
Average # of methods	3.5
Used ≥ 5 methods	49 (only 1 used 7)
Data analysis	
Thematic analysis	192
Field notes	123
Interview quotations	194
Survey results	60
Other	100
Overall design?	
Ethnographic	30
Participatory	6
Mixed/multi-methods	61
Evaluation	23
Qualitative	27
Case study	38
IS methods	
Standard IS outcomes	146
Use terms facilitators or barriers	159
Use implementation TMF?	144
CFIR	49
PARIHS/iPARIHS	13
PRECEDE-PROCEED	3
RE-AIM	4
Context	
Clinical setting	
Inpatient	62
Community	56
Outpatient (primary)	43
Outpatient (specialty)	32
Long term care	8
Emergency	7
Multiple	17
Unspecified	2
Country (top 5)	
United States of America	54
Canada	33
England	29
Australia	18
Sweden/China/Denmark	6

The included studies had considerable variation in the description of their overall design (Supplemental file 4); 29% of the manuscripts (*n* = 67) had some form of mixed methods (e.g., convergent parallel mixed methods, hybrid mixed methods, mixed methods time-motion study) as their study design. Many study designs were case studies (*n* = 46, 20%) and these also encompassed a broad range of qualifying descriptions, including qualitative, descriptive-explanatory case study; prospective observational case study; narrative case study; multiple case study with nested levels of analysis; and comparative, qualitative, explanatory embedded case study design. The descriptors were used in many different combinations as well. Ethnography (*n* = 34, 15%) including focused, institutional, rapid, interpretive, and autoethnography; evaluation (*n* = 33, 15%) including formative, mixed methods, process, prospective, realist, longitudinal, and partially mixed sequential dominant status evaluation; and participatory (*n* = 9) were common descriptions of study designs, although there were many others (e.g., critical pedagogy, situational analysis, quality improvement) and several (*n* = 14, 6%) did not include any study design description that could be identified by our team. Interestingly, eight studies (4%) described their design simply as qualitative. The variety and heterogeneity of terms and their combinations to describe research suggests a discomfort or mismatch between the realist epistemology of anthropological practice and the need to describe the work in terms considered methodologically rigorous.

### Hidden anthropology?

In answer to our primary research question about whether and how anthropology is practiced in implementation research, ethnography is not usually mentioned explicitly but components of the anthropological methodological toolkit often are. A third of the included texts (*n* = 73, 32%) mention ethnography explicitly with 30 (13%) describing the overall study design as ethnographic, yet only 18 (8%) mention anthropology either as a discipline or as the identity of one or more of the research team members. In anticipation of this invisibility or lack of explicit identification in terms of anthropological background, our inclusion criteria were based on the overall design, data collection, and analysis, where we felt that even “hidden” anthropology, which could be conceptualized more as an epistemic sensibility, would emerge with careful consideration.

**Fig. 2:**
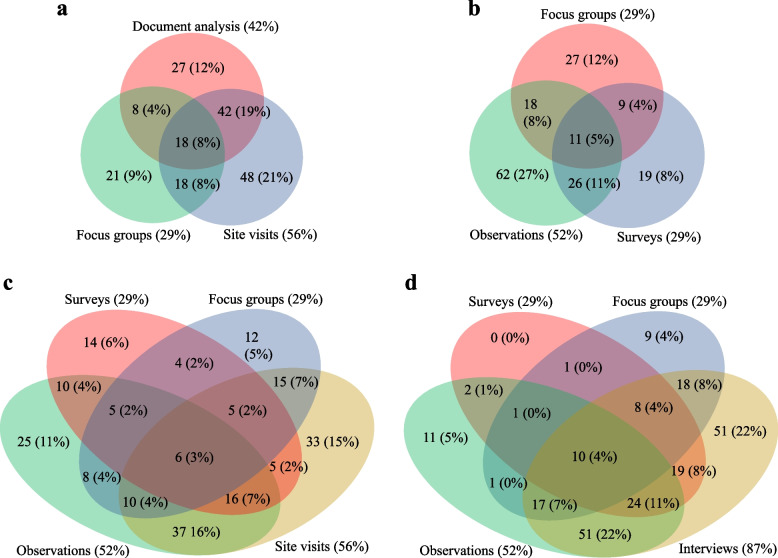
Co-occurrence of data collection methods. **a** Number and percentage of all articles (*n* = 227) reporting the use of document analysis, focus groups, and/or site visits. Forty-four articles (19%) reported no use of any of these methods. **b** Number and percentage of all articles (*n* = 227) reporting the use of focus groups, observations and/or surveys. Fifty-five articles (24%) reported no use of any of these methods. **c** Number and percentage of all articles (*n* = 227) reporting the use of surveys, focus groups, observations and/or site visits. Twenty-two articles (10%) reported no use of any of these methods. **d** Number and percentage of all articles (*n* = 227) reporting the use of surveys, focus groups, observations and/or interviews. Four articles (2%) reported no use of any of these methods

The individual data collection and analysis methods that compose ethnography are more frequent: 38% of the articles mention observation as a data collection method explicitly (*n* = 86), while another 70 articles (31%) imply observation from data collection methods such as site visits. Field notes, which tend to be the hallmark of long-term participant observation in anthropology, are used explicitly in most descriptions of data collection and analysis methods (*n* = 134, 59%), but those studies did not usually systematically describe findings from field notes in their results. The most often used method was some form of interview (i.e., structured, semi-structured, unstructured, informal) in both data collected (*n* = 198, 87%) and presentation of results, with 194 articles (85%) including direct quotations from the interviews. In general, most articles (*n* = 217, 96%) used multiple data collection and analytic methods, with 3.5 as the average number of specified data collection methods (not including “other methods”) and 20% of the articles using five or more methods (*n* = 49). The ones that used observation were more likely to have multiple data collection methods, as well (Fig. 2). Interestingly, only one used all seven data collection methods, and one was categorized as having described none of the traditional collection methods but was an auto-ethnography, which was written as a first-person narrative and described data collection as such.

**Fig. 3:**
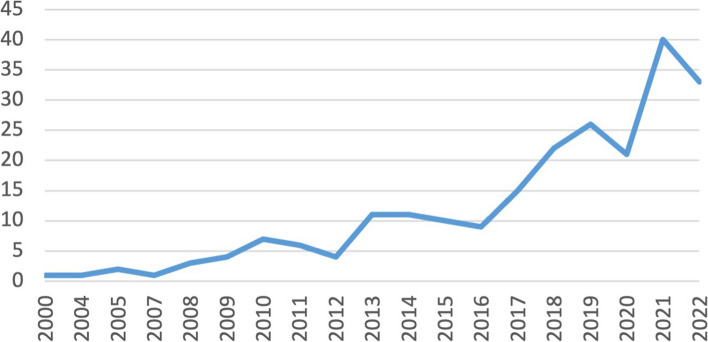
Number of articles included in review by year

**Fig. 4:**
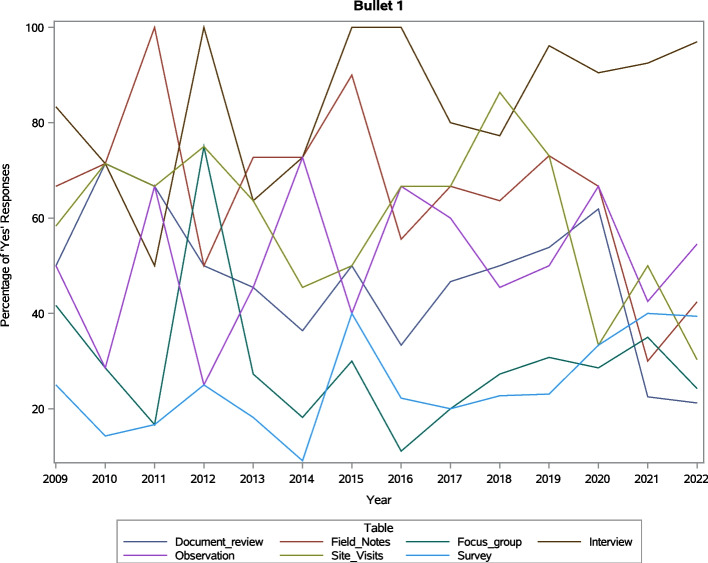
Frequency of data collection methods over time

The use of data collection methods described in the included manuscripts changed slightly over time. We did not include date limits in our searches, so the articles included in the results represent the duration of implementation science as a field and even a little before; the year of the first manuscript we included was published in 2000, and *Implementation Science*’s first issue was published in 2006. The number of included articles increased steadily over time until our final search in September 2022 (Fig. 3), which is not surprising given the growth of the field. The Cochrane-Armitrage Trend Test, which assesses whether there is an association between a two-level categorical variable (data collection methods) and an ordinal categorical variable (time) [24] however, gave us more details on individual data collection methods (Fig. 4). The analysis showed the frequency of observation and focus groups did not change significantly over time. Articles mentioning the use of interviews and surveys did increase over time, while field notes, document review, and site visits decreased over time.

Descriptions of analytic methods and techniques were more varied and were described using a wider variety of terminology overall. Most of the included articles mentioned some form of thematic or content analysis (*n* = 193, 85%), and more than half used field notes explicitly in the analysis or results sections (*n* = 123). However, descriptions of the overall analytic strategy, when explicit (12%, *n* = 28 included no description of an overarching analytic approach), showed the most heterogeneity. Those that did describe an analytic approach included mixed or multiple methods, deductive, inductive, grounded theory, triangulation, immersion crystallization, rapid ethnography, concept analysis, interpretive descriptive analysis, and framework analysis among others, and most articles described more than one analytic technique. Similar to the heterogeneity in study design descriptions, there was also no association between particular designs and analytic methods, although “qualitative” appeared most often across design, collection, and analysis domains.

### Does context matter?

One of our questions was whether anthropological approaches were used more often in one type or one aspect of implementation research. Our results show that research describing facilitators and barriers is represented in 70% of included manuscripts (*n* = 159), which aligns with our expectations that facilitators and barriers are more often investigated in implementation science with qualitative methods, as opposed to a topic in the anthropological literature. However, just slightly fewer (*n* = 146, 64%) described one or more standard implementation science outcomes (i.e., acceptability, adoption, feasibility, fidelity, reach, implementation cost, maintenance, and sustainability). The clinical setting was well distributed, although there were fewer studies conducted in long-term care and the emergency department, but that is likely reflective of overall implementation research. The top 4 countries where research was conducted were the USA, Canada, England, and Australia in rank order, but included studies encompassed 50 countries.

### Whose voice?

**Fig. 5:**
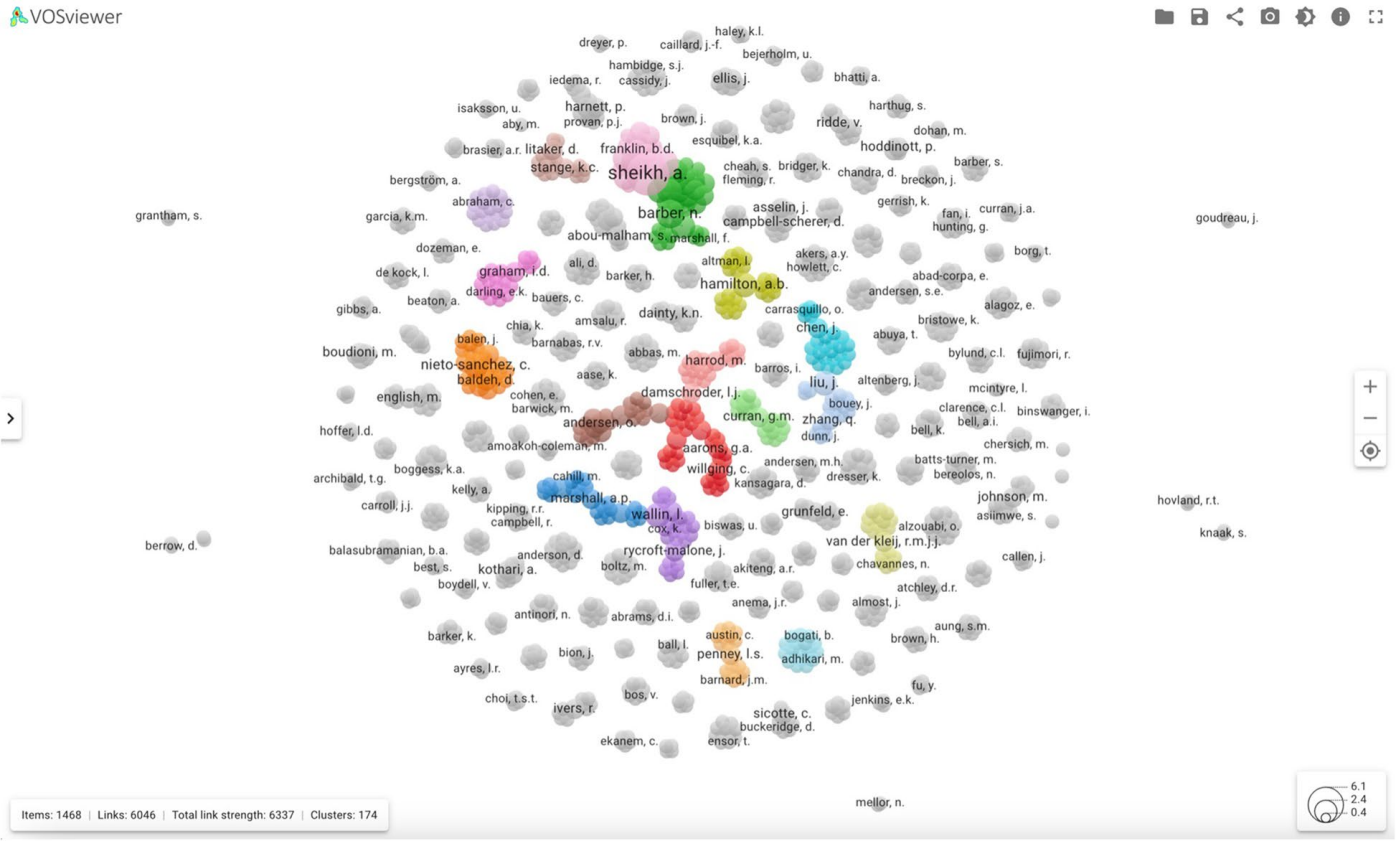
VOSViewer cluster density visualization

The bibliometric analyses conducted through Scopus, SciVal, and VOS Viewer contributed to an overall understanding of who is publishing this type of work, where, and the relationships between them. Figure 5 shows the cluster density visualization created by VOSViewer, and Table 2 shows details for the top 15 authors. The visualization shows co-authorship links between authors of the included publications; a *link* for each author represents the number of co-authors that person has in the dataset and the *total link strength* would be the number of co-authored publications that the author has in the dataset. Of the 15 authors who have the most links and strongest total links, only three are trained as anthropologists and two of those work for the US Department of Veteran Affairs (VA), which is known for its large number of medical anthropologist researchers [25]. The training disciplines of the others include public health, sociology, psychology, medicine, nursing, and computer and information science. Not all the top 15 were first authors on included publications, but their strong associations reflect the team science nature of implementation research.

**Table 2:** Details of the top 15 authors in the VOSViewer cluster density visualization

Author	Links	Total link strength	Documents	Clusters	Discipline
Sheihk, A	37	78	9	18	Medicine/Epidemiology
Barber, N	29	50	4	2	Pharmacy
Liu, J	35	35	3	12	Public Health
Nieto-Sanchez, C	24	32	3	7	Medical Anthropology
Damschroder, L	28	28	2	10	Public Health
Chen, J	26	26	2	6	Computer & Information Science
Hamilton, A	26	26	3	4	Anthropology
Wallin, L	25	26	3	5	Nursing
Rycroft-Malone, J	21	21	2	5	Nursing/Psychology
Graham, I.D	21	21	2	9	Medical Sociology
Harrod, M	20	20	2	10	Anthropology
Van der Klej, R	19	19	2	13	Psychology
Curran, G	19	19	2	11	Medical Sociology
Penney, L.S	17	17	2	16	Anthropology
Anderson, B. O	12	15	2	8	Medicine

While only 220 articles were included in the Scopus analysis because seven did not have a PMID, Scopus is considered a trustworthy bibliometric data source for research assessments, research landscape studies, science policy evaluations, and university rankings [26]. The top 5 journals in which included articles were published were *BMC Health Services Research* (*n* = 22), *Implementation Science* (*n* = 14), *BMJ Open* (*n* = 7), *BMJ Quality and Safety* (*n* = 6), and *Social Science and Medicine* (*n* = 6). The included 220 articles were published across 121 unique journals. The top 4 funding sources listed for the research were the Canadian Institute of Health Research (*n* = 16), National Institute for Health Research (*n* = 14), National Institutes for Health (*n* = 22), and the National Health and Medical Research Council (*n* = 7) of 158 funding sources captured in Scopus. Scopus also analyzes the affiliations of the authors, the top 5 of which were the University of Toronto (*n* = 11), University of Edinburgh (*n* = 10), University of Alberta (*n* = 10), University of Sydney (*n* = 8), and University of Montreal (*n* = 7), but there were 159 institutional affiliations listed overall.

## Discussion

To place our work within the broader context of work focused on methodological and theoretical approaches in implementation science, the first step is looking at the related scoping reviews by Gertner et al. and Hagaman et al. All three adopted the same methodological frame of a ‘scoping’ review [27, 28], with similar research questions around the use of methodological approaches in implementation science. Their scoping reviews approached analyses deductively, with pre-determined definitions of ethnography and qualitative methods, respectively. Our current work approached the review inductively, in which we allowed the results of our bibliographic search to inform our characterization of the field iteratively as we progressed [29]. Gertner et al.’s review criteria focused specifically on articles that used the term ‘ethnography’ in implementation science research, with 73 articles included in their final analysis, while Hagaman et al.’s was extremely broad and looked at qualitative approaches in implementation research, which resulted in 867 articles included in their qualitative synthesis.

Their different approaches led to very different results; Gertner’s review concluded with recommendations for how researchers might better describe the use of ethnographic methods in implementation research (i.e., researcher training and position, researchers’ positionality, detailed description of observational methods, and inclusion of all results). The main relevant takeaways from the Hagaman review were that given inconsistencies in descriptions of analytic method(s) and variations in transparency of design choices, detailed guidelines may increase the rigorous integration of qualitative methods into theoretically informed implementation research.

Our scoping review fell somewhere in the middle, both in terms of the breadth and depth of question and search strategy and in terms of our results and takeaways. This likely reflects our inductive approach, in which we iteratively interrogated what the overarching goals of our study were and what we hoped to have as the takeaway. We began with the idea that we wanted to document how ethnography was used in implementation science; the publication of Gertner’s review shortly after we began challenged us to broaden our conceptualization of ethnography—a conversation that continues even now. The presentation of Hagaman’s results close to the end of our review process helped us clarify that were not solely concerned with the documentation of which suites of qualitative methods are being used in implementation science. In fact, through our process we ended up seeing both the epistemic sensibility and methodological approaches as necessary for something to be “ethnographic”—so deploying a set of methods is not really a guarantee of ethnographically sensitive results. More broadly, the heterogeneity in our results reflects a dissonance between the goals of anthropological inquiry and practice and the ways in which this work is described in the literature. Most included studies used qualifying descriptions of their study designs and analytic methods to make the unstructured work of anthropological practice fit into neatly defined terms.

In our analysis of the results of the data abstraction, it was clear that our process reflected our team’s decisions, identities, and relationships with implementation science and our prioritization to remain as close to the text as possible [27]. Despite our standardized data extraction worksheet, we did not achieve consistency in every aspect of our review. For example, we collected the overall analytic approach but not in a way that we could neatly characterize how many articles used which approach, given the overlap and breadth of descriptors for both (as an example, just the analytic approach of case study included five types: qualitative, descriptive-explanatory case study; prospective observational case study; narrative case study; multiple case study with nested levels of analysis; and comparative, qualitative, explanatory embedded case study design. Our screening and data abstraction processes and the results produced reflect the tension between concerns of perceived methodological protectionism [12], while also wanting to demonstrate that anthropology is being practiced in implementation science work despite its disciplinary invisibility.

There was a constant hesitation to define boundaries and therefore canonize our own interpretations of how ethnography is or should be used in implementation research. This led to the bibliographic and network analyses, which allowed us to investigate who is driving the way anthropological approaches are being written and whether or not the articles we chose to include represented anthropologists practicing in implementation research. In fact, one critical insight from the overall process is that our conventional way of referring to ethnography as method tends to miss the point that it comes with an epistemic viewpoint, and when we lose that, the methods lose much of their meaning and value. Another way to answer these questions could be a qualitative study of anthropologists in implementation research. For example, anthropologists working in the Veterans Health Administration represent a big portion of practitioners in this space [25], yet it was difficult to “see” them in the way we analyzed affiliations or funding sources.

Our initial hypothesis that anthropologists’ work within implementation research is often invisible is reflected in our results, although our own positionality may have biased that result. Anthropological work often supports larger mixed-methods implementation projects without being explicitly or thoroughly reported. Building on Gertner’s results, we found the use of characteristic tools of ethnography like field notes often is not systematically described (in a way recognizable to other anthropologists) but is often mentioned. Further, we found that research design decisions and compromises (e.g., length of time in the field, logistics of stakeholder involvement, reconciling diverse firsthand experiences in team ethnography) that are integral to anthropological approaches are not systematically described. This may reflect the tradition within health services research, and to a lesser extent implementation science specifically, of deference to quantitative science. Within this bibliographic context, qualitative work is often erroneously construed as purely “descriptive”. Additionally, even though context is explicitly and implicitly fundamental to implementation research, the goals of ethnographic work and their relationship to larger research projects or institutional goals are often not explicitly stated. Without more explicit attention to the anthropological epistemology, implementation science may be missing out on insights anthropology offers on power dynamics; intersectional identities and diverse experiences; and embedded, structural, and systemic aspects of health and healthcare of different contexts.

## Limitations

This scoping review is inherently limited by our positionality and by the review process itself (i.e., incompatibility of defining ethnography for inclusion and exclusion). The dearth of ‘anthropology’ as a term in the literature may be a result of who is conducting the research, but it was unclear from our searches whether most authors were trained or practicing anthropologists. Additionally, our results were limited by our inclusion criteria; the top 4 countries (i.e., USA, Canada, England, and Australia) may be explained by our inclusion of English-only papers rather than as a reflection of where anthropological approaches are being used in implementation science. Future research could consider similar questions about anthropology in implementation science looking at the training and employment of the researchers directly rather than implying it from the descriptions of their work within the literature. Fix and colleagues have done similar work focused on the VA specifically [25, 30]. Nonetheless, our findings offer an important look into the role of anthropological methods in the implementation science literature as a way for practitioners to interrogate their own roles within the field and reflect on how they contribute to the canonization of ways of doing and publishing implementation research.

## Conclusions

Implementation science reflects complex organizational and behavioral change in diverse and equally complex contexts. Anthropology is well-suited and essential for implementation research to attend to the power dynamics; intersectional identities and diverse experiences; and embedded, structural, and systemic aspects of health and healthcare of the contexts in which we work. Given that history and epistemology inform current practice, this large, anthropological infusion likely has implications for how implementation science is practiced especially its attention to context. However, our review points to the challenges of trying to summarize a methodology that is creative and context-specific by nature. Over the course of this review process, we ourselves began to conceptualize our own anthropological practice in our implementation research differently and describe it more explicitly, both in grant proposals and in published manuscripts. From our own reflections, researchers doing qualitative work in implementation research could think critically about how their work is ethnographic from a methodological and epistemological standpoint to capture the richness of the ethnographic sensibility. More broadly, researchers doing implementation science might consider interrogating the disciplinary roots of their approach and how that informs all aspects of their work.

### Supplementary Information

**Additional file 1: Supplemental file 1.** Search Strategies. Search Strategies for PubMed, Embase, Cochrane CENTRAL, CINAHL, PsycINFO, Web of Science, and Anthropology Plus databases.

**Additional file 2: Supplemental file 2.** Abstract screening tool. Ethnography in Implementation Science Abstract Screening Inclusion Rubric for Second Round Screening.

**Additional file 3: Supplemental file 3.** Included articles. Citations for all 227 articles included in the full-text analysis.

**Additional file 4: Supplemental file 4.** Select attributes table. Table describing select attributes of included articles (i.e., year, explicit use of anthropology/ethnography, clinical setting, country, and overall design)

## Data Availability

The dataset used and analyzed during the current study are available from the corresponding author on reasonable request.
