# Supplementary material for: Implicit and explicit: a scoping review exploring the contribution of anthropological practice in implementation science
Source: Implement Sci. 2024 Feb 12;19:12. doi: 10.1186/s13012-024-01344-0 (PMC10863116; doi:10.1186/s13012-024-01344-0)
Supplement: Supplementary file 1 — Additional file 1: Supplemental file 1. Search Strategies. Search Strategies for PubMed, Embase, Cochrane CENTRAL, CINAHL, PsycINFO, Web of Science, and Anthropology Plus databases. [file 13012_2024_1344_MOESM1_ESM.docx]

**Search Strategies**

**PubMed**

(("Anthropology, Cultural"[Mesh] OR "Anthropology, Medical"[Mesh] OR anthropology[tw] OR ethnography[tw] OR ethnographies[tw] OR ethnographic[tw] OR ethno graphic[tw] OR anthropologist[tw] OR anthropologists[tw] OR anthropologist s[tw] OR rapid assessment[tw] OR rapid assessments[tw] OR immersion crystallization[tw] OR immersion crystallisation[tw] OR participant observation[tw] OR participant observations[tw] OR reflexivity[tw] OR emic[tw] OR site visit[tw] OR site visits[tw] OR onsite visit[tw] OR onsite visits[tw] OR "Interviews as Topic"[Mesh] OR interview as topic[tw] OR interviews as topic[tw] OR indepth interview[tw] OR indepth interviews[tw] OR indepth interviewing[tw] OR in depth interview[tw] OR in depth interviews[tw] OR in depth interviewing[tw] OR semistructured interview[tw] OR semistructured interviews[tw] OR semistructured interviewing[tw] OR semi structured interview[tw] OR semi structured interviews[tw] OR semi structured interviewing[tw]) AND ("Implementation Science"[Mesh] OR implementation science[tw] OR implementation sciences[tw] OR implementation scientist[tw] OR implementation scientists[tw] OR improvement science[tw] OR improvement sciences[tw] OR implementation research[tw] OR implementation framework[tw] OR implementation frameworks[tw] OR implementation model[tw] OR implementation models[tw] OR implementation strategy[tw] OR implementation strategies[tw] OR knowledge translation[tiab] OR knowledge translations[tiab] OR knowledge transfer[tiab] OR knowledge transfers[tiab] OR intervention implementation[tw] OR ((healthcare practices[tw] OR health care practices[tw]) AND implementation[tw]))) NOT ((((("Systematic Review" [Publication Type]) OR "Meta-Analysis" [Publication Type]) OR "Letter" [Publication Type]) OR "Editorial" [Publication Type]) OR "Comment" [Publication Type])

**Embase**

#37. #36 NOT ('letter'/exp OR 'editorial'/exp OR 'conference paper'/exp OR 'systematic review'/exp)

#36. #20 AND #35

#35. #21 OR #22 OR #23 OR #24 OR #25 OR #26 OR #27 OR #28 OR #29 OR #30 OR #31 OR #32 OR #33 OR #34

#34. (('healthcare practices' OR 'health care practices') NEAR/3 implementation):ti,ab,kw

#33. 'intervention implementation$':ti,ab,kw

#32. 'knowledge transfer$':ti,ab,kw

#31. 'knowledge translation$':ti,ab,kw

#30. 'implementation strateg*':ti,ab,kw

#29. 'implementation model$':ti,ab,kw

#28. 'implementation framework$':ti,ab,kw

#27. 'implementation research':ti,ab,kw

#26. 'implementation scientific research':ti,ab,kw

#25. 'improvement science$':ti,ab,kw

#24. 'implementation scientist$':ti,ab,kw

#23. 'implementation science$':ti,ab,kw

#22. 'implementation scientist'/exp

#21. 'implementation science'/exp

#20. #1 OR #2 OR #3 OR #4 OR #5 OR #6 OR #7 OR #8 OR #9 OR #10 OR #11 OR #12 OR #13 OR #14 OR #15 OR #16 OR #17 OR #18 OR #19

#19. 'semi structured interview'/exp

#18. 'semistructured interview*':ti,ab,kw

#17. 'semi structured interview*':ti,ab,kw

#16. 'indepth interview*':ti,ab,kw

#15. 'in depth interview*':ti,ab,kw

#14. 'interview$ as topic':ti,ab,kw

#13. 'onsite visit$':ti,ab,kw

#12. 'site visit$':ti,ab,kw

#11. emic:ti,ab,kw

#10. reflexivity:ti,ab,kw

#9. 'participant observation$":ti,ab,kw

#8. 'immersion crystalli?ation':ti,ab,kw

#7. 'rapid assessment$':ti,ab,kw

#6. ethnograph*:ti,ab,kw OR 'ethno graphic':ti,ab,kw

#5. anthropologist*:ti,ab,kw

#4. anthropology:ti,ab,kw

#3. 'medical anthropology'/exp

#2. 'cultural anthropology'/exp

#1. 'ethnography'/exp

**Cochrane CENTRAL**

#1 MeSH descriptor: [Anthropology, Cultural] explode all trees

#2 MeSH descriptor: [Anthropology, Medical] explode all trees

#3 (anthropology):ti,ab,kw

#4 (anthropologist*):ti,ab,kw

#5 (ethnograph*):ti,ab,kw

#6 (("ethno graphic")):ti,ab,kw

#7 ("rapid assessment*"):ti,ab,kw

#8 ("immersion crystalli?ation"):ti,ab,kw

#9 ("participant observation" OR "participant observations"):ti,ab,kw

#10 (reflexivity):ti,ab,kw

#11 (emic):ti,ab,kw

#12 ("site visit" OR "site visits"):ti,ab,kw

#13 ("onsite visit" OR "onsite visits"):ti,ab,kw

#14 MeSH descriptor: [Interviews as Topic] explode all trees

#15 ("interview as topic" OR "interviews as topic"):ti,ab,kw

#16 ("indepth interview" OR "indepth interviews" OR "indepth interviewing"):ti,ab,kw

#17 ("in-depth interview" OR "in-depth interviews" OR "in-depth interviewing"):ti,ab,kw

#18 ("semistructured interview" OR "semistructured interviews" OR "semistructured interviewing"):ti,ab,kw

#19 ("semi-structured interview" OR "semi-structured interviews" OR "semi-structured interviewing"):ti,ab,kw

#20 {OR #1-#19}

#21 MeSH descriptor: [Implementation Science] explode all trees

#22 ("implementation science" OR "implementation sciences"):ti,ab,kw

#23 ("implementation scienctist" OR "implementation scientists"):ti,ab,kw

#24 ("improvement science" OR "improvement sciences"):ti,ab,kw

#25 ("implementation scientific research"):ti,ab,kw

#26 ("implementation research"):ti,ab,kw

#27 ("implementation framework" OR "implementation frameworks"):ti,ab,kw

#28 (("implementation model" OR "implementation models")):ti,ab,kw

#29 (("implementation strategy" OR "implementation strategies")):ti,ab,kw

#30 ("knowledge transfer" OR "knowledge transfers"):ti,ab,kw

#31 ("knowledge translation" OR "knowledge translations"):ti,ab,kw

#32 ("intervention implementation" OR "intervention implementations"):ti,ab,kw

#33 (("healthcare practices" OR "health care practices") NEAR/3 implementation):ti,ab,kw

#34 {OR #21-#33}

#35 #20 AND #34

**CINAHL**

S10 S4 AND S9

S9 S5 OR S6 OR S7 OR S8

S8 AB anthropology OR ethnograph* OR “ethno graphic” OR anthropologist* OR “rapid assessment*” OR “immersion crystalli?ation" OR “participant observation*” OR reflexivity OR emic OR “site visit*” OR “onsite visit*” OR “interview* as topic” OR “indepth interview*” OR “in depth interview*” OR “semistructured interview*” OR “semi structured interview*”

S7 TI anthropology OR ethnograph* OR “ethno graphic” OR anthropologist* OR “rapid assessment*” OR “immersion crystalli?ation" OR “participant observation*” OR reflexivity OR emic OR “site visit*” OR “onsite visit*” OR “interview* as topic” OR “indepth interview*” OR “in depth interview*” OR “semistructured interview*” OR “semi structured interview*”

S6 MH "Semi-Structured Interview"

S5 MH "Anthropology, Cultural"

S4 S1 OR S2 OR S3

S3 AB “implementation science*” OR “implementation scientist*” OR “improvement science*” OR “implementation research” OR “implementation framework*” OR “implementation model*” OR “implementation strateg*” OR “knowledge transfer*” OR “knowledge translation*” OR “intervention implementation*” OR ((“healthcare practices” OR “health care practices”) N3 implementation))

S2 TI “implementation science*” OR “implementation scientist*” OR “improvement science*” OR “implementation research” OR “implementation framework*” OR “implementation model*” OR “implementation strateg*” OR “knowledge transfer*” OR “knowledge translation*” OR “intervention implementation*” OR ((“healthcare practices” OR “health care practices”) N3 implementation))

S1 MH "Implementation Science"

**PsycINFO**

S12 S6 AND S10 AND S11

S11 health OR healthcare OR "care delivery" OR medical OR medicine OR biomedical OR biomedicine OR "bio medicine" OR nursing OR "primary care" OR rehabilitation OR pediatricians OR "assisted living"

S10 S7 OR S8 OR S9

S9 KW “implementation science*” OR “implementation scientist*” OR “improvement science*” OR “implementation research” OR “implementation framework*” OR “implementation model*” OR “implementation strateg*” OR “knowledge transfer*” OR “knowledge translation*” OR “intervention implementation*” OR ((“healthcare practices” OR “health care practices”) N3 implementation)

S8 AB “implementation science*” OR “implementation scientist*” OR “improvement science*” OR “implementation research” OR “implementation framework*” OR “implementation model*” OR “implementation strateg*” OR “knowledge transfer*” OR “knowledge translation*” OR “intervention implementation*” OR ((“healthcare practices” OR “health care practices”) N3 implementation)

S7 TI “implementation science*” OR “implementation scientist*” OR “improvement science*” OR “implementation research” OR “implementation framework*” OR “implementation model*” OR “implementation strateg*” OR “knowledge transfer*” OR “knowledge translation*” OR “intervention implementation*” OR ((“healthcare practices” OR “health care practices”) N3 implementation)

S6 S1 OR S2 OR S3 OR S4 OR S5

S5 KW anthropology OR ethnograph* OR “ethno graphic” OR anthropologist* OR “rapid assessment*” OR “immersion crystalli?ation" OR “participant observation*” OR reflexivity OR emic OR “site visit*” OR “onsite visit*” OR “interview* as topic” OR “indepth interview*” OR “in depth interview*” OR “semistructured interview*” OR “semi structured interview*”

S4 AB anthropology OR ethnograph* OR “ethno graphic” OR anthropologist* OR “rapid assessment*” OR “immersion crystalli?ation" OR “participant observation*” OR reflexivity OR emic OR “site visit*” OR “onsite visit*” OR “interview* as topic” OR “indepth interview*” OR “in depth interview*” OR “semistructured interview*” OR “semi structured interview*”

S3 TI anthropology OR ethnograph* OR “ethno graphic” OR anthropologist* OR “rapid assessment*” OR “immersion crystalli?ation" OR “participant observation*” OR reflexivity OR emic OR “site visit*” OR “onsite visit*” OR “interview* as topic” OR “indepth interview*” OR “in depth interview*” OR “semistructured interview*” OR “semi structured interview*”

S2 DE "Semi-Structured Interview"

S1 DE "Ethnography"

**Web of Science**

13 #12 AND #8 AND #4

12 #11 OR #10 OR #9

11 AK=(health OR healthcare OR "care delivery" OR medical OR medicine OR biomedical OR biomedicine OR "bio medicine" OR nursing OR "primary care" OR rehabilitation OR pediatricians OR "assisted living")

10 AB=(health OR healthcare OR "care delivery" OR medical OR medicine OR biomedical OR biomedicine OR "bio medicine" OR nursing OR "primary care" OR rehabilitation OR pediatricians OR "assisted living")

9 TI=(health OR healthcare OR "care delivery" OR medical OR medicine OR biomedical OR biomedicine OR "bio medicine" OR nursing OR "primary care" OR rehabilitation OR pediatricians OR "assisted living")

8 #7 OR #6 OR #5

7 AK=(anthropology OR ethnograph* OR “ethno graphic” OR anthropologist* OR “rapid assessment*” OR “immersion crystalli*ation" OR “participant observation*” OR reflexivity OR emic OR “site visit*” OR “onsite visit*” OR “interview* as topic” OR “indepth interview*” OR “in depth interview*” OR “semistructured interview*” OR “semi structured interview*”)

6 AB=(anthropology OR ethnograph* OR “ethno graphic” OR anthropologist* OR “rapid assessment*” OR “immersion crystalli*ation" OR “participant observation*” OR reflexivity OR emic OR “site visit*” OR “onsite visit*” OR “interview* as topic” OR “indepth interview*” OR “in depth interview*” OR “semistructured interview*” OR “semi structured interview*”)

5 TI=(anthropology OR ethnograph* OR “ethno graphic” OR anthropologist* OR “rapid assessment*” OR “immersion crystalli*ation" OR “participant observation*” OR reflexivity OR emic OR “site visit*” OR “onsite visit*” OR “interview* as topic” OR “indepth interview*” OR “in depth interview*” OR “semistructured interview*” OR “semi structured interview*”)

4 #3 OR #2 OR #1

3 AK=("implementation science*" OR "implementation scientist*" OR "improvement science*" OR "implementation research" OR "implementation framework*" OR "implementation model*" OR "implementation strateg*" OR “knowledge transfer*” OR “knowledge translation*” OR "translational research" OR "translational science" OR "translational medicine" OR "translational medical" OR "translational clinical" OR "intervention implementation*" OR (("healthcare practices" OR "health care practices") NEAR/3 implementation))

2 AB=("implementation science*" OR "implementation scientist*" OR "improvement science*" OR "implementation research" OR "implementation framework*" OR "implementation model*" OR "implementation strateg*" OR “knowledge transfer*” OR “knowledge translation*” OR "translational research" OR "translational science" OR "translational medicine" OR "translational medical" OR "translational clinical" OR "intervention implementation*" OR (("healthcare practices" OR "health care practices") NEAR/3 implementation))

1 TI=("implementation science*" OR "implementation scientist*" OR "improvement science*" OR "implementation research" OR "implementation framework*" OR "implementation model*" OR "implementation strateg*" OR “knowledge transfer*” OR “knowledge translation*” OR "translational research" OR "translational science" OR "translational medicine" OR "translational medical" OR "translational clinical" OR "intervention implementation*" OR (("healthcare practices" OR "health care practices") NEAR/3 implementation))

**Anthropology Plus**

S3 S1 AND S2

S2 anthropology OR ethnograph* OR “ethno graphic” OR anthropologist* OR “rapid assessment*” OR “immersion crystalli?ation" OR “participant observation*” OR reflexivity OR emic OR “site visit*” OR “onsite visit*” OR “interview* as topic” OR “indepth interview*” OR “in depth interview*” OR “semistructured interview*” OR “semi structured interview*”

S1 “implementation science*” OR “implementation scientist*” OR “improvement science*” OR “implementation research” OR “implementation framework*” OR “implementation model*” OR “implementation strateg*” OR “knowledge transfer*” OR “knowledge translation*” OR “intervention implementation*” OR ((“healthcare practices” OR “health care practices”) N3 implementation))
