# Supplementary material for: Implicit and explicit: a scoping review exploring the contribution of anthropological practice in implementation science
Source: Implement Sci. 2024 Feb 12;19:12. doi: 10.1186/s13012-024-01344-0 (PMC10863116; doi:10.1186/s13012-024-01344-0)
Supplement: Supplementary file 2 — Additional file 2: Supplemental file 2. Abstract screening tool. Ethnography in Implementation Science Abstract Screening Inclusion Rubric for Second Round Screening. [file 13012_2024_1344_MOESM2_ESM.pdf]

Ethnography in Implementation Science Abstract Screening Inclusion Rubric

Second Round Screening

Citation, Title, and Abstract Screening

1. Is the article in English?
   a. Yes: Include
   b. No: Exclude
2. Is the article original research?
   a) Yes or unclear: investigate further, continue screening
   b) No: exclude **and mark in the Label box "Not original research"**
3. Is the article about a health-focused intervention?
   1. Yes or unclear: investigate further, continue screening
   2. No: exclude **and mark in the Label box "Not health-focused intervention"**

4. Is it an abstract, or protocol, methods, review, or policy paper?

   1. No or unclear: investigate further, continue screening
   2. Yes: exclude **and mark in the Label box "Abstract", "Protocol Paper", "Methods Paper", "Review" or "Protocol Paper" as appropriate**

5. Is it operationally "ethnographic" ("anthropology" or "ethnography" specifically mentioned OR some combination of: multiple methods (NOT including unrelated 1 QUAL + 1 QUANT), theoretical/methodological, field notes, observation, triangulation, ethnography, immersion crystallization, site visits, comparative study, multiple perspectives, in situ, iterative, longitudinal, participatory, reflexivity)?

   1. Yes or unclear: investigate further, continue screening
   2. No: exclude **and mark in the Label box "Not ethnographic"**

6. Is it operationally "implementation science" ("implementation science/research" or "knowledge translation" specifically mentioned OR some combination of an IS theory/model/framework, stage of implementation (preparation, implementation, etc.), implementation outcomes, potentially facilitator/barriers with something else)?

    1. Yes or unclear: investigate further, continue screening
    2. No: exclude **and mark in the Label box "Not IS"**

7. Is there another reason it shouldn't be included?

    1. No or unclear: investigate further, continue screening
    2. Yes: exclude **and mark in the Label box "Other" and add brief reason for exclusion in the Note box**

8. If title/abstract passes all of the above, **include for full review**
