# Supplementary material for: Implicit and explicit: a scoping review exploring the contribution of anthropological practice in implementation science
Source: Implement Sci. 2024 Feb 12;19:12. doi: 10.1186/s13012-024-01344-0 (PMC10863116; doi:10.1186/s13012-024-01344-0)
Supplement: Supplementary file 3 — Additional file 3: Supplemental file 3. Included articles. Citations for all 227 articles included in the full-text analysis. [file 13012_2024_1344_MOESM3_ESM.docx]

**Full List of 227 Included Articles**

1. Abad‐Corpa E, Delgado‐Hito P, Cabrero‐García J, Meseguer‐Liza C, Zárate‐Riscal CL, Carrillo‐Alcaraz A, et al. Implementing evidence in an onco‐haematology nursing unit: a process of change using participatory action research. International Journal of Evidence‐Based Healthcare. 2013;11(1):46-55.

2. Abou Malham S, Smithman M-A, Touati N, Brousselle A, Loignon C, Dubois C-A, et al. Explaining variation of implementation outcomes of centralized waiting lists for unattached patients: A comparative multiple case study in Quebec. Journal of Health Organization and Management. 2019;33(5):563-87.

3. Abou Malham S, Touati N, Maillet L, Gaboury I, Loignon C, Breton M. What are the factors influencing implementation of advanced access in family medicine units? A cross-case comparison of four early adopters in Quebec. International journal of family medicine. 2017;2017.

4. Abou-Malham S, Hatem M, Leduc N. Analyzing barriers and facilitators to the implementation of an action plan to strengthen the midwifery professional role: a Moroccan case study. BMC health services research. 2015;15:1-15.

5. Aby M. A case study of implementing grant-funded integrated care in a community mental health center. The journal of behavioral health services & research. 2020;47(2):293-308.

6. Adeoye-Olatunde OA, Curran GM, Jaynes HA, Hillman LA, Sangasubana N, Chewning BA, et al. Preparing for the spread of patient-reported outcome (PRO) data collection from primary care to community pharmacy: a mixed-methods study. Implementation Science Communications. 2022;3(1):1-17.

7. Amoakoh-Coleman M, Ansah E, Klipstein-Grobusch K, Arhinful D. Completeness of obstetric referral letters/notes from subdistrict to district level in three rural districts in Greater Accra region of Ghana: an implementation research using mixed methods. BMJ open. 2019;9(9):e029785.

8. Anastasaki M, van Bree EM, Brakema EA, Tsiligianni I, Sifaki-Pistolla D, Chatzea VE, et al. Beliefs, Perceptions, and Behaviors Regarding Chronic Respiratory Diseases of Roma in Crete, Greece: A Qualitative FRESH AIR Study. Frontiers in Public Health. 2022;10:781.

9. Andersen MH, Urstad KH, Larsen MH, Engebretsen E, Ødemark J, Eriksen A, et al. Processes of knowing in the translation of a health communication intervention for dialysis patients awaiting kidney transplantation. Patient Education and Counseling. 2021;104(3):517-23.

10. Asselin J, Osunlana A, Ogunleye A, Sharma A, Campbell‐Scherer D. Missing an opportunity: the embedded nature of weight management in primary care. Clinical obesity. 2015;5(6):325-32.

11. Austin EJ, Briggs ES, Ferro L, Barry P, Heald A, Curran GM, et al. Integrating routine screening for opioid use disorder into primary care settings: Experiences from a national cohort of clinics. Journal of General Internal Medicine. 2023;38(2):332-40.

12. Balis LE, Adjei KK, Nyame S, Opoku Mensah J, Asante KP. Stakeholder perspectives on adapting and disseminating Ghana’s physical activity guidelines: a qualitative study. BMC public health. 2021;21:1-12.

13. Baral S, Subedi HN, Paudel P, Chand PB, Shrestha MP, McCullough A, et al. Implementation research to assess a health workers performance-based management system in Nepal. Acta Paediatr. 2018;107 Suppl 471:24-34.

14. Bardwell G, Strike C, Altenberg J, Barnaby L, Kerr T. Implementation contexts and the impact of policing on access to supervised consumption services in Toronto, Canada: a qualitative comparative analysis. Harm Reduction Journal. 2019;16(1):1-9.

15. Beaton A, Manuel C, Tapsell J, Foote J, Oetzel JG, Hudson M. He Pikinga Waiora: supporting Māori health organisations to respond to pre-diabetes. International Journal for Equity in Health. 2019;18:1-11.

16. Belaid L, Benoit M, Kaur N, Lili A, Ridde V. Population health intervention implementation among migrants with precarious status in Montreal: Underlying theory and key challenges. SAGE open. 2020;10(2):2158244020917957.

17. Best S, Long JC, Braithwaite J, Taylor N. Standardizing variation: Scaling up clinical genomics in Australia. Genetics in Medicine. 2023;25(2):100109.

18. Bos V, Roorda D, De Sonnaville E, Van Boven M, Oosterlaan J, van Goudoever J, et al. Implementing structured follow-up of neonatal and paediatric patients: an evaluation of three university hospital case studies using the functional resonance analysis method. BMC health services research. 2022;22(1):191.

19. Bosma AR, Boot CR, Schaap R, Schaafsma FG, Anema JR. Participatory Approach to create a supportive work environment for employees with chronic conditions: a pilot implementation study. Journal of Occupational and Environmental Medicine. 2022;64(8):665.

20. Boudioni M, McLaren S, Lister G. Patient empowerment: Its implementation and systems within hospitals in England and Greece. Health Services Management Research. 2018;31(4):180-94.

21. Boydell V, Neema S, Wright K, Hardee K. Closing the Gap between People and Programs: Lessons from Implementation of Social Accountability for Family Planning and Reproductive Health in Uganda. Afr J Reprod Health. 2018;22(1):73-84.

22. Bridges J, May C, Fuller A, Griffiths P, Wigley W, Gould L, et al. Optimising impact and sustainability: a qualitative process evaluation of a complex intervention targeted at compassionate care. BMJ quality & safety. 2017;26(12):970-7.

23. Brunton L, Sammut-Powell C, Birleson E, Boaden R, Knowles SE, McQuaker C, et al. Scale-up of ABC care bundle for intracerebral haemorrhage across two hyperacute stroke units in one region in England: a mixed methods evaluation of a quality improvement project. BMJ Open Quality. 2022;11(2):e001601.

24. Burton W, Twiddy M, Sahota P, Brown J, Bryant M. Participant engagement with a UK community-based preschool childhood obesity prevention programme: a focused ethnography study. BMC public health. 2019;19:1-14.

25. Byrnes A, Young A, Mudge A, Banks M, Clark D, Bauer J. Prospective application of an implementation framework to improve postoperative nutrition care processes: Evaluation of a mixed methods implementation study. Nutrition & Dietetics. 2018;75(4):353-62.

26. Cammer A, Morgan D, Stewart N, McGilton K, Rycroft-Malone J, Dopson S, et al. The hidden complexity of long-term care: how context mediates knowledge translation and use of best practices. The Gerontologist. 2014;54(6):1013-23.

27. Campbell R, Rawlins E, Wells S, Kipping RR, Chittleborough CR, Peters TJ, et al. Intervention fidelity in a school-based diet and physical activity intervention in the UK: Active for Life Year 5. International Journal of Behavioral Nutrition and Physical Activity. 2015;12(1):1-14.

28. Carroll JJ, Mackin S, Schmidt C, McKenzie M, Green TC. The Bronze Age of drug checking: barriers and facilitators to implementing advanced drug checking amidst police violence and COVID-19. Harm reduction journal. 2022;19(1):1-13.

29. Chenoweth L, Jeon Y-H, Stein-Parbury J, Forbes I, Fleming R, Cook J, et al. PerCEN trial participant perspectives on the implementation and outcomes of person-centered dementia care and environments. International Psychogeriatrics. 2015;27(12):2045-57.

30. Choi T, Walker K, Lombard C, Palermo C. Optimising the effectiveness of diabetes education in an East Asian population. Nutrition & Dietetics. 2017;74(3):253-60.

31. Clack L, Zingg W, Saint S, Casillas A, Touveneau S, da Liberdade Jantarada F, et al. Implementing infection prevention practices across European hospitals: an in-depth qualitative assessment. BMJ quality & safety. 2018;27(10):771-80.

32. Cole CB, Pacca J, Mehl A, Tomasulo A, van der Veken L, Viola A, et al. Toward communities as systems: a sequential mixed methods study to understand factors enabling implementation of a skilled birth attendance intervention in Nampula Province, Mozambique. Reprod Health. 2018;15(1):132.

33. Cope EL, Johnson M, Khan M, Kaplan HC, Sales A, Mistry KB. Contextual factors affecting implementation of pediatric quality improvement programs. Academic pediatrics. 2022;22(3):S81-S91.

34. Cordasco KM, Zuchowski JL, Hamilton AB, Kirsh S, Veet L, Saavedra JO, et al. Early lessons learned in implementing a women's health educational and virtual consultation program in VA. Medical care. 2015;53(4 Suppl 1):S88.

35. Courtney-Pratt H, Cummings E, Turner P, Cameron-Tucker H, Wood-Baker R, Walters EH, et al. Entering a world of uncertainty: community nurses’ engagement with information and communication technology. CIN: Computers, Informatics, Nursing. 2012;30(11):612-9.

36. Cresswell K, Morrison Z, Crowe S, Robertson A, Sheikh A. Anything but engaged: user involvement in the context of a national electronic health record implementation. Informatics in Primary Care. 2011;19(4).

37. Cresswell K, Sheikh A, Franklin BD, Krasuska M, The Nguyen H, Hinder S, et al. Interorganizational knowledge sharing to establish digital health learning ecosystems: qualitative evaluation of a national digital health transformation program in England. Journal of medical Internet research. 2021;23(8):e23372.

38. Cresswell KM, Mozaffar H, Lee L, Williams R, Sheikh A. Workarounds to hospital electronic prescribing systems: a qualitative study in English hospitals. BMJ quality & safety. 2017;26(7):542-51.

39. Cresswell KM, Worth A, Sheikh A. Integration of a nationally procured electronic health record system into user work practices. BMC medical informatics and decision making. 2012;12:1-12.

40. Dainty KN, Golden BR, Hannam R, Webster F, Browne G, Mittmann N, et al. A realist evaluation of value-based care delivery in home care: the influence of actors, autonomy and accountability. Social Science & Medicine. 2018;206:100-9.

41. Dainty KN, Racz E, Morrison LJ, Brooks SC. Implementation of a post-arrest care team: understanding the nuances of a team-based intervention. Implementation Science. 2015;11(1):1-8.

42. Dale E, Conigrave KM, Kelly PJ, Ivers R, Clapham K, Lee KK. A Delphi yarn: applying Indigenous knowledges to enhance the cultural utility of SMART Recovery Australia. Addiction Science & Clinical Practice. 2021;16:1-15.

43. Damush T, Plue L, Schmid A, Anderson J, Murphy C, Kimmel B, et al. Adapting secondary stroke prevention programs to local resources, structures and front line inputs. Stroke. 2009;40(4):e280.

44. Davis M, Balasubramanian BA, Waller E, Miller BF, Green LA, Cohen DJ. Integrating behavioral and physical health care in the real world: early lessons from advancing care together. The Journal of the American Board of Family Medicine. 2013;26(5):588-602.

45. Davis SM, Cruz TH, Kozoll RL. Research to practice: implementing physical activity recommendations. American journal of preventive medicine. 2017;52(3):S300-S3.

46. DePue JD, Rosen RK, Batts-Turner M, Bereolos N, House M, Held RF, et al. Cultural translation of interventions: diabetes care in American Samoa. American journal of public health. 2010;100(11):2085-93.

47. Dipankui MT. Collaboration between researchers and knowledge users in health technology assessment: a qualitative exploratory study. International Journal of Health Policy and Management. 2017;6(8):437.

48. Dlamini-Simelane T, Moyer E. Task shifting or shifting care practices? The impact of task shifting on patients’ experiences and health care arrangements in Swaziland. BMC health services research. 2017;17(1):1-12.

49. Dmitrieva A, Stepanov V, Lukash I-G, Martynyuk A. Performance indicator as the main and the only goal: A “dark side” of the intervention aims to accelerate HIV treatment entry among people who inject drugs in Kyiv, Ukraine. Harm Reduction Journal. 2019;16(1):1-11.

50. Doran D, Paterson J, Clark C, Srivastava R, Goering PN, Kushniruk AW, et al. A pilot study of an electronic interprofessional evidence‐based care planning tool for clients with mental health problems and addictions. Worldviews on Evidence‐Based Nursing. 2010;7(3):174-84.

51. Downey J, Shearn K, Brown N, Wadey R, Breckon J. Behaviour change practices in exercise referral schemes: developing realist programme theory of implementation. BMC health services research. 2021;21(1):1-9.

52. Draper CE, Nemutandani SM, Grimsrud AT, Rudolph M, Kolbe-Alexander TL, de Kock L, et al. Qualitative evaluation of a physical activity-based chronic disease prevention program in a low-income, rural South African setting. Rural and Remote Health. 2010;10(3):1-14.

53. Drew S, Judge A, Cohen R, Fitzpatrick R, Barker K, Gooberman-Hill R. Enhanced recovery after surgery implementation in practice: an ethnographic study of services for hip and knee replacement. BMJ open. 2019;9(3):e024431.

54. Dunn J, Zhang Q, Weeks MR, Li J, Liao S, Li F. Indigenous hiv prevention beliefs and practices among low-earning Chinese sex workers as context for introducing female condoms and other novel prevention options. Qualitative health research. 2017;27(9):1302-15.

55. Eckstrom E, Neal MB, Cotrell V, Casey CM, McKenzie G, Morgove MW, et al. An interprofessional approach to reducing the risk of falls through enhanced collaborative practice. Journal of the American Geriatrics Society. 2016;64(8):1701-7.

56. Egerton T, Lawford BJ, Campbell PK, Plinsinga ML, Spiers L, Mackenzie DA, et al. Expert-moderated peer-to-peer online support group for people with knee osteoarthritis: mixed methods randomized controlled pilot and feasibility study. JMIR Formative Research. 2022;6(1):e32627.

57. El Oussoul S, Haesebaert J, Leaune E, Haesebaert F. From Knowledge Transfer to Action: An Example of a Community of Practice for First-Episode Psychosis in Lyon, France. Psychiatric Services. 2020;71(9):975-8.

58. Elliott SA, Wright KS, Scott SD, Hartling L. Perspectives From French and Filipino Parents on the Adaptation of Child Health Knowledge Translation Tools: Qualitative Exploration. JMIR Formative Research. 2022;6(3):e33156.

59. Ellis J, Band R, Kinsella K, Cheetham-Blake T, James E, Ewings S, et al. Optimising and profiling pre-implementation contexts to create and implement a public health network intervention for tackling loneliness. Implement Sci. 2020;15(1):35.

60. Ellis J, Vassilev I, James E, Rogers A. Implementing a social network intervention: can the context for its workability be created? A quasi-ethnographic study. Implementation Science Communications. 2020;1:1-11.

61. English M, Nzinga J, Mbindyo P, Ayieko P, Irimu G, Mbaabu L. Explaining the effects of a multifaceted intervention to improve inpatient care in rural Kenyan hospitals-interpretation based on retrospective examination of data from participant observation, quantitative and qualitative studies. Implementation Science. 2011;6:1-12.

62. Erichsen Andersson A, Frödin M, Dellenborg L, Wallin L, Hök J, Gillespie BM, et al. Iterative co-creation for improved hand hygiene and aseptic techniques in the operating room: experiences from the safe hands study. BMC health services research. 2018;18(1):1-12.

63. Etokidem A, Nkpoyen F, Ekanem C, Mpama E, Isika A. Potential barriers to and facilitators of civil society organization engagement in increasing immunization coverage in Odukpani Local Government Area of Cross River State, Nigeria: an implementation research. Health Research Policy and Systems. 2021;19(2):1-12.

64. Ezumah N, Manzano A, Ezenwaka U, Obi U, Ensor T, Etiaba E, et al. Role of trust in sustaining provision and uptake of maternal and child healthcare: evidence from a national programme in Nigeria. Social Science & Medicine. 2022;293:114644.

65. Fassier J-B, Durand M-J, Caillard J-F, Roquelaure Y, Loisel P. Results of a feasibility study: barriers and facilitators in implementing the Sherbrooke model in France. Scandinavian Journal of Work, Environment & Health. 2015:223-33.

66. Fehr A, Muela J, Nieto-Sanchez C, Manneh E, Baldeh D, Ceesay O, et al. The role of social cohesion in the implementation and coverage of a mass drug administration trial for malaria control in the Gambia: An in-depth comparison of two intervention villages. Social Science & Medicine. 2021;291:114487.

67. Fehr A, Nieto-Sanchez C, Muela J, Jaiteh F, Ceesay O, Maneh E, et al. From informed consent to adherence: factors influencing involvement in mass drug administration with ivermectin for malaria elimination in The Gambia. Malar J. 2021;20(1):198.

68. Fernandes BD, Foppa AA, Ayres LR, Chemello C. Implementation of Medication Reconciliation conducted by hospital pharmacists: A case study guided by the Consolidated Framework for Implementation Research. Research in Social and Administrative Pharmacy. 2022;18(9):3631-7.

69. Folker AP, Mathiasen K, Lauridsen SM, Stenderup E, Dozeman E, Folker MP. Implementing internet-delivered cognitive behavior therapy for common mental health disorders: A comparative case study of implementation challenges perceived by therapists and managers in five European internet services. Internet Interventions. 2018;11:60-70.

70. Forchuk C, Martin ML, Jensen E, Ouseley S, Sealy P, Beal G, et al. Integrating an evidence‐based intervention into clinical practice:‘Transitional relationship model’. Journal of Psychiatric and Mental Health Nursing. 2013;20(7):584-94.

71. Forman J, Harrod M, Robinson C, Annis-Emeott A, Ott J, Saffar D, et al. First things first: foundational requirements for a medical home in an academic medical center. Journal of general internal medicine. 2014;29:640-8.

72. Fu Y, Wang C, Hu Y, Muir‐Cochrane E. The barriers to evidence‐based nursing implementation in mainland China: A qualitative content analysis. Nursing & health sciences. 2020;22(4):1038-46.

73. Fujimori R, Liu K, Soeno S, Naraba H, Ogura K, Hara K, et al. Acceptance, barriers, and facilitators to implementing artificial intelligence–based decision support systems in emergency departments: quantitative and qualitative evaluation. JMIR formative research. 2022;6(6):e36501.

74. Fuller TE, Pong DD, Piniella N, Pardo M, Bessa N, Yoon C, et al. Interactive digital health tools to engage patients and caregivers in discharge preparation: Implementation study. Journal of Medical Internet Research. 2020;22(4).

75. Gallant N, Hadjistavropoulos T, Winters EM, Feere EK, Wickson-Griffiths A. Development, evaluation, and implementation of an online pain assessment training program for staff in rural long-term care facilities: a case series approach. BMC geriatrics. 2022;22(1):336.

76. Garney WR, Szucs LE, Primm K, King Hahn L, Garcia KM, Martin E, et al. Implementation of policy, systems, and environmental community-based interventions for cardiovascular health through a national not-for-profit: a multiple case study. Health Education & Behavior. 2018;45(6):855-64.

77. Georgiou A, Greenfield T, Callen J, Westbrook JI. Safety and efficiency considerations for the introduction of electronic ordering in a blood bank. Archives of pathology & laboratory medicine. 2009;133(6):933-7.

78. Gerrish K, Laker S, Taylor C, Kennedy F, McDonnell A. Enhancing the quality of oral nutrition support for hospitalized patients: a mixed methods knowledge translation study (The EQONS study). Journal of Advanced Nursing. 2016;72(12):3182-94.

79. Gibbs A, Jewkes R, Mbatha N, Washington L, Willan S. Jobs, food, taxis and journals: complexities of implementing Stepping Stones and Creating Futures in urban informal settlements in South Africa. Afr J AIDS Res. 2014;13(2):161-7.

80. Goedken CC, Livorsi DJ, Sauder M, Vander Weg MW, Chasco EE, Chang N-C, et al. “The role as a champion is to not only monitor but to speak out and to educate”: the contradictory roles of hand hygiene champions. Implementation Science. 2019;14(1):1-11.

81. Goicolea I, Coe AB, Hurtig AK, San Sebastian M. Mechanisms for achieving adolescent-friendly services in Ecuador: a realist evaluation approach. Glob Health Action. 2012;5.

82. Goldman J, Meuser J, Lawrie L, Rogers J, Reeves S. Interprofessional primary care protocols: a strategy to promote an evidence-based approach to teamwork and the delivery of care. Journal of interprofessional care. 2010;24(6):653-65.

83. Grealish L, Chaboyer W, Mudge A, Simpson T, Cahill M, Todd JA, et al. Using a general theory of implementation to plan the introduction of delirium prevention for older people in hospital. Journal of Nursing Management. 2019;27(8):1631-9.

84. Greenland K, Chipungu J, Chilekwa J, Chilengi R, Curtis V. Disentangling the effects of a multiple behaviour change intervention for diarrhoea control in Zambia: a theory-based process evaluation. Globalization and health. 2017;13(1):1-18.

85. Guan TH, Htut HN, Davison CM, Sebastian S, Bartels SA, Aung SM, et al. Implementation of a neonatal hepatitis B immunization program in rural Karenni State, Myanmar: A mixed-methods study. PLoS One. 2021;16(12):e0261470.

86. Guise A, Ndimbii J, Igonya EK, Owiti F, Strathdee SA, Rhodes T. Integrated and differentiated methadone and HIV care for people who use drugs: a qualitative study in Kenya with implications for implementation science. Health policy and planning. 2019;34(2):110-9.

87. Gupta M, Rahman A, Dutta N, Nambiar D, Ivers R, Jagnoor J. Opportunities for gender transformative approaches in a community-based drowning reduction program in Bangladesh. International journal for equity in health. 2020;19:1-11.

88. Hagiwara MA, Suserud BO, Jonsson A, Henricson M. Exclusion of context knowledge in the development of prehospital guidelines: results produced by realistic evaluation. Scand J Trauma Resusc Emerg Med. 2013;21:46.

89. Halleberg Nyman M, Forsman H, Wallin L, Ostaszkiewicz J, Hommel A, Eldh AC. Promoting evidence-based urinary incontinence management in acute nursing and rehabilitation care-A process evaluation of an implementation intervention in the orthopaedic context. J Eval Clin Pract. 2019;25(2):282-9.

90. Hamilton AB, Cohen AN, Glover DL, Whelan F, Chemerinski E, McNagny KP, et al. Implementation of evidence‐based employment services in specialty mental health. Health services research. 2013;48(6pt2):2224-44.

91. Harmon TG, Hardy L, Haley KL. Proactive social validation of methods and procedures used for training speech production in aphasia. Aphasiology. 2018;32(8):922-43.

92. Harrison D, Wilson R, Graham A, Brown K, Hesselgreaves H, Ciesielska M. Making every contact count with seldom‐heard groups? A qualitative evaluation of voluntary and community sector (VCS) implementation of a public health behaviour change programme in England. Health & Social Care in the Community. 2022;30(5):e3193-e206.

93. Harrison MB, Graham ID, van den Hoek J, Dogherty EJ, Carley ME, Angus V. Guideline adaptation and implementation planning: a prospective observational study. Implementation science. 2013;8:1-14.

94. Harrison MI, Grantham S. Learning from implementation setbacks: Identifying and responding to contextual challenges. Learning health systems. 2018;2(4):e10068.

95. Harsha Bangura A, Nirola I, Thapa P, Citrin D, Belbase B, Bogati B, et al. Measuring fidelity, feasibility, costs: an implementation evaluation of a cluster-controlled trial of group antenatal care in rural Nepal. Reproductive health. 2020;17(1):1-12.

96. Haun JN, Lind JD, Shimada SL, Martin TL, Gosline RM, Antinori N, et al. Evaluating user experiences of the secure messaging tool on the Veterans Affairs' patient portal system. Journal of Medical Internet Research. 2014;16(3):266-81.

97. Haverhals LM, Gilman C, Manheim C, Bauers C, Kononowech J, Levy C. Implementation of VA's Life-Sustaining Treatment Decisions Initiative: Facilitators and Barriers to Early Implementation Across Seven VA Medical Centers. J Pain Symptom Manage. 2021.

98. Hennein R, Ggita J, Ssuna B, Shelley D, Akiteng AR, Davis JL, et al. Implementation, interrupted: Identifying and leveraging factors that sustain after a programme interruption. Global Public Health. 2022;17(9):1868-82.

99. Henry D, Wood S, Moshashane N, Ramontshonyana K, Amutah C, Maleki P, et al. Facilitators and barriers to implementation of long-acting reversible contraceptive services for adolescent girls and young women in Gaborone, Botswana. Journal of pediatric and adolescent gynecology. 2021;34(4):504-13.

100. Hien D, Kaboré JMT, Siribié M, Soulama I, Barry N, Baguiya A, et al. Stakeholder perceptions on the deployment of multiple first-line therapies for uncomplicated malaria: A qualitative study in the health district of Kaya, Burkina Faso. Malaria Journal. 2022;21(1):202.

101. Higgins A, Murphy R, Downes C, Varley J, Begley C, Elliott N. Factors influencing the implementation of epilepsy specialist nurse role: using the consolidation framework for implementation research. Journal of Clinical Nursing. 2020;29(7-8):1352-64.

102. Hinckley JJ. A case for the implementation of cognitive-communication screenings in acute stroke. 2014.

103. Hinder S, Cresswell K, Sheikh A, Franklin BD, Krasuska M, The Nguyen H, et al. Promoting inter-organisational knowledge sharing: a qualitative evaluation of England’s Global Digital Exemplar and Fast Follower Programme. Plos one. 2021;16(8):e0255220.

104. Hoddinott P, Britten J, Pill R. Why do interventions work in some places and not others: a breastfeeding support group trial. Social science & medicine. 2010;70(5):769-78.

105. Hoddinott P, Pill R, Chalmers M. Health professionals, implementation and outcomes: reflections on a complex intervention to improve breastfeeding rates in primary care. Family practice. 2007;24(1):84-91.

106. Holloway B, Mathur A, Pathak A, Bergstrom A. Utilisation of diagnostics in India: a rapid ethnographic study exploring context and behaviour. BMJ Open. 2020;10(11):e041087.

107. Holm A, Nikolajsen L, Dreyer P. A multicomponent intervention to optimise nurse–patient communication in the intensive care unit: A mixed-methods acceptability and feasibility study. Australian Critical Care. 2022;35(6):616-22.

108. Hovland RT, Moltu C. Making way for a clinical feedback system in the narrow space between sessions: Navigating competing demands in complex healthcare settings. International Journal of Mental Health Systems. 2019;13:1-11.

109. Howard R, Delaney L, Kilbourne AM, Kidwell KM, Smith S, Englesbe M, et al. Development and implementation of preoperative optimization for high-risk patients with abdominal wall hernia. JAMA Network Open. 2021;4(5):e216836-e.

110. Hsu C, Liss DT, Westbrook EO, Arterburn D. Incorporating patient decision aids into standard clinical practice in an integrated delivery system. Medical Decision Making. 2013;33(1):85-97.

111. Humphrey C, Berrow D. In the eye of the beholder: Problems of perception in designing a strategy to promote evidence-based clinical policy. Journal of Evaluation in Clinical Practice. 2000;6(2):165-76.

112. Hunting G, Shahid N, Sahakyan Y, Fan I, Moneypenny CR, Stanimirovic A, et al. A multi-level qualitative analysis of Telehomecare in Ontario: challenges and opportunities. BMC health services research. 2015;15(1):1-15.

113. Husain A, Cohen E, Dubrowski R, Jamieson T, Kurahashi AM, Lokuge B, et al. A clinical communication tool (loop) for team-based care in pediatric and adult care settings: hybrid mixed methods implementation study. Journal of Medical Internet Research. 2021;23(3):e25505.

114. Husser EK, Fick DM, Boltz M, Shrestha P, Siuta J, Malloy S, et al. Implementing a Rapid, Two-Step Delirium Screening Protocol in Acute Care: Barriers and Facilitators. J Am Geriatr Soc. 2021;69(5):1349-56.

115. Indraratna P, Biswas U, Liu H, Redmond SJ, Yu J, Lovell NH, et al. Process Evaluation of a Randomised Controlled Trial for TeleClinical Care, a Smartphone-App Based Model of Care. Frontiers in Medicine. 2022;8:780882.

116. Irimu GW, Greene A, Gathara D, Kihara H, Maina C, Mbori-Ngacha D, et al. Explaining the uptake of paediatric guidelines in a Kenyan tertiary hospital–mixed methods research. BMC health services research. 2014;14:1-13.

117. Iyengar K, Gupta M, Pal S, Kaur K, Singla N, Verma M, et al. Baseline Assessment of Evidence-Based Intrapartum Care Practices in Medical Schools in 3 States in India: A Mixed-Methods Study. Global Health: Science and Practice. 2022;10(2).

118. Jabbour M, Newton AS, Johnson D, Curran JA. Defining barriers and enablers for clinical pathway implementation in complex clinical settings. Implementation Science. 2018;13(1):1-13.

119. Jackson GL, Cutrona SL, White BS, Reardon CM, Orvek E, Nevedal AL, et al. Merging implementation practice and science to scale up promising practices: the veterans health administration (VHA) diffusion of excellence (DoE) program. The Joint Commission Journal on Quality and Patient Safety. 2021;47(4):217-27.

120. Jacobsen FF, Mekki TE, Førland O, Folkestad B, Kirkevold Ø, Skår R, et al. A mixed method study of an education intervention to reduce use of restraint and implement person-centered dementia care in nursing homes. BMC nursing. 2017;16:1-11.

121. Janssen A, Robinson T, Brunner M, Harnett P, Museth KE, Shaw T. Multidisciplinary teams and ICT: a qualitative study exploring the use of technology and its impact on multidisciplinary team meetings. BMC health services research. 2018;18:1-10.

122. Johanson S, Markström U, Larsson ME, Bejerholm U. Implementation of a novel return-to-work approach for persons with affective disorders in a traditional vocational rehabilitation context: a case study. International Journal of Mental Health Systems. 2020;14:1-12.

123. Jones LK, Hayduk V, Romagnoli KM, Flango A, Marks J, Graham J, et al. Evaluation of an educational strategy to improve medication reconciliation in ambulatory care. Journal of the American Pharmacists Association. 2022;62(4):1179-88. e3.

124. Jürgens G, Jacobsen C, Rasmussen H, Werge T, Nordentoft M, Andersen S. Utility and adoption of CYP2D6 and CYP2C19 genotyping and its translation into psychiatric clinical practice. Acta psychiatrica scandinavica. 2012;125(3):228-37.

125. Kandasami S, Shobiye H, Fakoya A, Asiimwe S, Inimah M, Etukoit M, et al. Can changes in service delivery models improve program quality and efficiency? A closer look at HIV programs in Kenya and Uganda. Journal of Acquired Immune Deficiency Syndromes (1999). 2019;81(5):533.

126. Karltun A, Sanne JM, Aase K, Anderson JE, Fernandes A, Fulop NJ, et al. Knowledge management infrastructure to support quality improvement: A qualitative study of maternity services in four European hospitals. Health Policy. 2020;124(2):205-15.

127. Kavle JA, Picolo M, Buccini G, Barros I, Dillaway CH, Pérez-Escamilla R. Strengthening counseling on barriers to exclusive breastfeeding through use of job aids in Nampula, Mozambique. PLoS One. 2019;14(12):e0224939.

128. Kerrigan V, Kelly A, Lee AM, Mungatopi V, Mitchell AG, Wyber R, et al. A community-based program to reduce acute rheumatic fever and rheumatic heart disease in northern Australia. BMC health services research. 2021;21:1-15.

129. Kettlewell J, Radford K, Kendrick D, Patel P, Bridger K, Kellezi B, et al. Qualitative study exploring factors affecting the implementation of a vocational rehabilitation intervention in the UK major trauma pathway. BMJ open. 2022;12(3):e060294.

130. Kirk JW, Nilsen P, Andersen O, Stefánsdóttir NT, Grønfeldt B, Brødsgaard R, et al. Adaptations and modifications to a co-designed intervention and its clinical implementation: a qualitative study in Denmark. BMC health services research. 2021;21(1):1108.

131. Kirk JW, Stefánsdóttir NÞ, Powell BJ, Lindstroem MB, Andersen O, Tjørnhøj-Thomsen T, et al. Oilcloth sessions as an implementation strategy: a qualitative study in Denmark. BMC Medical Education. 2022;22(1):571.

132. Knaak S, Patten S. A grounded theory model for reducing stigma in health professionals in Canada. Acta Psychiatrica Scandinavica. 2016;134:53-62.

133. Kotarba JA, Wooten K, Freeman J, Brasier AR. The culture of translational science research: participants’ stories. International Review of Qualitative Research. 2013;6(1):127-42.

134. Kothari A, Boyko JA, Conklin J, Stolee P, Sibbald SL. Communities of practice for supporting health systems change: a missed opportunity. Health Research Policy and Systems. 2015;13(1):1-9.

135. Kristensen HK, Borg T, Hounsgaard L. Facilitation of Research-Based Evidence within Occupational Therapy in Stroke Rehabilitation. British Journal of Occupational Therapy. 2011;74(10):473-83.

136. Kristiansen M, Nedergaard Jensen A, Norredam M, Srivarathan A. Targeting preventive home visits to older adults in disadvantaged communities: Perspectives of professionals. Health & Social Care in the Community. 2021;29(4):1051-60.

137. Lafort Y, Ismael de Melo MS, Lessitala F, Griffin S, Chersich M, Delva W. Feasibility, acceptability and potential sustainability of a ‘diagonal’approach to health services for female sex workers in Mozambique. BMC health services research. 2018;18(1):1-11.

138. Langlois S, Goudreau J. “From Health Experts to Health Guides”: Motivational Interviewing Learning Processes and Influencing Factors. Health education & behavior. 2022:10901981221084271.

139. Latif A, Waring J, Watmough D, Barber N, Chuter A, Davies J, et al. Examination of England's New Medicine Service (NMS) of complex health care interventions in community pharmacy. Research in Social and Administrative Pharmacy. 2016;12(6):966-89.

140. Lengnick-Hall R, Willging C, Hurlburt M, Fenwick K, Aarons GA. Contracting as a bridging factor linking outer and inner contexts during EBP implementation and sustainment: a prospective study across multiple US public sector service systems. Implementation Science. 2020;15:1-16.

141. Lennox L, Barber S, Stillman N, Spitters S, Ward E, Marvin V, et al. Conceptualising interventions to enhance spread in complex systems: a multisite comprehensive medication review case study. BMJ Quality & Safety. 2022;31(1):31-44.

142. Leppin AL, Boehmer KR, Branda ME, Shah ND, Hargraves I, Dick S, et al. Developing a toolkit to implement the Statin Choice Conversation Aid at scale: application of a work reduction model. BMC health services research. 2019;19(1):249.

143. Lewis NV, Dowrick A, Sohal A, Feder G, Griffiths C. Implementation of the Identification and Referral to Improve Safety programme for patients with experience of domestic violence and abuse: A theory-based mixed-method process evaluation. Health Soc Care Community. 2019;27(4):e298-e312.

144. Lim RBT, Cheung ON, Tham DKT, La HH, Win TT, Chan R, et al. Using qualitative and community-based engagement approaches to gain access and to develop a culturally appropriate STI prevention intervention for foreign female entertainment workers in Singapore. Globalization and health. 2018;14(1):1-12.

145. Litaker D, Ruhe M, Flocke S. Making sense of primary care practices’ capacity for change. Translational Research. 2008;152(5):245-53.

146. Liu J, Ponzer S, Farrokhnia N, Masiello I. Evaluation of interprofessional teamwork modules implementation in an emergency department–A mixed-methods case study of implementation fidelity. BMC health services research. 2021;21(1):853.

147. Luig T, Asselin J, Sharma AM, Campbell-Scherer DL. Understanding implementation of complex interventions in primary care teams. The Journal of the American Board of Family Medicine. 2018;31(3):431-44.

148. Manhas KP, Olson K, Churchill K, Vohra S, Wasylak T. Implementation of a novel rehabilitation model of care across Alberta, Canada: a focused ethnography. BMJ Open Quality. 2021;10(1):e001261.

149. Marshall AP, Tobiano G, Murphy N, Comadira G, Willis N, Gardiner T, et al. Handover from operating theatre to the intensive care unit: A quality improvement study. Australian Critical Care. 2019;32(3):229-36.

150. Masunaga Y, Jaiteh F, Manneh E, Balen J, Okebe J, D'Alessandro U, et al. The community lab of ideas for health: community-based transdisciplinary solutions in a malaria elimination trial in The Gambia. Frontiers in public health. 2021;9:637714.

151. McCarthy MS, Ujano-De Motta LL, Nunnery MA, Gilmartin H, Kelley L, Wills A, et al. Understanding adaptations in the Veteran Health Administration's Transitions Nurse Program: refining methodology and pragmatic implications for scale-up. Implement Sci. 2021;16(1):71.

152. McCullough MB, Chou AF, Solomon JL, Petrakis BA, Kim B, Park AM, et al. The interplay of contextual elements in implementation: an ethnographic case study. BMC health services research. 2015;15:62.

153. McKay VR, Margaret Dolcini M, Hoffer LD. The dynamics of de-adoption: a case study of policy change, de-adoption, and replacement of an evidence-based HIV intervention. Transl Behav Med. 2017;7(4):821-31.

154. McLaren S, Woods L, Boudioni M, Lemma F, Tavabie A. Implenting a strategy to promote lifelong learning in the primary care workforce: An evaluation of leadership roles, change management approaches, interim challenges and achievements. Quality in Primary Care. 2008;16(3):147-55.

155. Melder A, Robinson T, Mcloughlin I, Iedema R, Teede H. Integrating the complexity of healthcare improvement with implementation science: a longitudinal qualitative case study. BMC health services research. 2022;22(1):1-12.

156. Mellor N, Webster J. Enablers and challenges in implementing a comprehensive workplace health and well‐being approach. International Journal of Workplace Health Management. 2013;6(2):129-42.

157. Moffat BM, Jenkins EK, Johnson JL. Weeding out the information: an ethnographic approach to exploring how young people make sense of the evidence on cannabis. Harm Reduction Journal. 2013;10:1-9.

158. Mølgaard RR, Jørgensen L, Christensen EF, Grønkjær M, Voldbjerg SL. Ambivalence in nurses’ use of the early warning score: A focussed ethnography in a hospital setting. Journal of advanced nursing. 2022;78(5):1461-72.

159. Moore SK, Saunders EC, McLeman B, Metcalf SA, Walsh O, Bell K, et al. Implementation of a New Hampshire community-initiated response to the opioid crisis: a mixed-methods process evaluation of Safe Station. International Journal of Drug Policy. 2021;95:103259.

160. Mosch LK, Poncette A-S, Spies C, Weber-Carstens S, Schieler M, Krampe H, et al. Creation of an Evidence-Based Implementation Framework for Digital Health Technology in the Intensive Care Unit: Qualitative Study. JMIR Formative Research. 2022;6(4):e22866.

161. Motulsky A, Sicotte C, Moreault M-P, Schuster T, Girard N, Buckeridge D, et al. Using Health Information Exchange: Usage and Perceived Usefulness in Primary Care...17th World Congress on Medical and Health Informatics, August 25-30, 2019, Lyon, France. Studies in Health Technology & Informatics. 2019;264:709-13.

162. Mozaffar H, Cresswell KM, Williams R, Bates DW, Sheikh A. Exploring the roots of unintended safety threats associated with the introduction of hospital ePrescribing systems and candidate avoidance and/or mitigation strategies: a qualitative study. BMJ quality & safety. 2017;26(9):722-33.

163. Mumtaz Z, Levay A, Bhatti A, Salway S. Good on paper: the gap between programme theory and real‐world context in Pakistan's Community Midwife programme. BJOG: An International Journal of Obstetrics & Gynaecology. 2015;122(2):249-58.

164. Munro J, McIntyre L. (Not) getting political: indigenous women and preventing mother-to-child transmission of HIV in West Papua. Cult Health Sex. 2016;18(2):157-72.

165. Murdoch J, Varley A, McCulloch J, Jones M, Thomas LB, Clark A, et al. Implementing supportive exercise interventions in the colorectal cancer care pathway: a process evaluation of the PREPARE-ABC randomised controlled trial. BMC cancer. 2021;21:1-12.

166. Murphy JK, Michalak EE, Liu J, Colquhoun H, Burton H, Yang X, et al. Barriers and facilitators to implementing measurement-based care for depression in Shanghai, China: a situational analysis. BMC Psychiatry. 2021;21(1):430.

167. Ninemire MR, Petrescu M, Musick MA, Shekerdemian LS, Kumar S, Dinh J, et al. Lessons and Insights From the Implementation of the Cohort Model for a PICU: A Case Study Research. Pediatric Critical Care Medicine. 2022;23(10):e456-e64.

168. O'Malley G, Asrat L, Sharma A, Hamunime N, Stephanus Y, Br, et al. Nurse task shifting for antiretroviral treatment services in Namibia: implementation research to move evidence into action. PLoS One. 2014;9(3):e92014.

169. Or C, Dohan M, Tan J. Understanding critical barriers to implementing a clinical information system in a nursing home through the lens of a socio-technical perspective. J Med Syst. 2014;38(9):99.

170. Paciocco S, Kothari A, Licskai CJ, Ferrone M, Sibbald SL. Evaluating the implementation of a chronic obstructive pulmonary disease management program using the Consolidated Framework for Implementation Research: a case study. BMC health services research. 2021;21(1):1-13.

171. Palacio A, Keller VF, Chen J, Tamariz L, Carrasquillo O, Tanio C. Can Physicians Deliver Chronic Medications at the Point of Care? Am J Med Qual. 2016;31(3):256-64.

172. Parkes T, Carver H, Masterton W, Booth H, Ball L, Murdoch H, et al. Exploring the potential of implementing managed alcohol programmes to reduce risk of COVID-19 infection and transmission, and wider harms, for people experiencing alcohol dependency and homelessness in Scotland. International Journal of Environmental Research and Public Health. 2021;18(23):12523.

173. Penney LS, Bharath PS, Miake-Lye I, Leng M, Olmos-Ochoa TT, Finley EP, et al. Toolkit and distance coaching strategies: a mixed methods evaluation of a trial to implement care coordination quality improvement projects in primary care. BMC health services research. 2021;21(1):817.

174. Pérez MC, Ch, ra D, Koné G, Singh R, Ridde V, et al. Implementation fidelity and acceptability of an intervention to improve vaccination uptake and child health in rural India: a mixed methods evaluation of a pilot cluster randomized controlled trial. Implement Sci Commun. 2020;1:88.

175. Pinto C, Bristowe K, Witt J, Davies JM, de Wolf-Linder S, Dawkins M, et al. Perspectives of patients, family caregivers and health professionals on the use of outcome measures in palliative care and lessons for implementation: a multi-method qualitative study. Ann Palliat Med. 2018;7(Suppl 3):S137-S50.

176. Power R, Ussher JM, Hawkey A, Missiakos O, Perz J, Ogunsiji O, et al. Co-designed, culturally tailored cervical screening education with migrant and refugee women in Australia: a feasibility study. BMC Women's Health. 2022;22(1):1-17.

177. Pusa S, Isaksson U, Sundin K. Evaluation of the implementation process of a family systems nursing approach in home health care: a mixed-methods study. Journal of Family Nursing. 2021;27(3):235-49.

178. Quanbeck A, Brown RT, Zgierska AE, Jacobson N, Robinson JM, Johnson RA, et al. A randomized matched-pairs study of feasibility, acceptability, and effectiveness of systems consultation: a novel implementation strategy for adopting clinical guidelines for opioid prescribing in primary care. Implementation Science. 2018;13:1-13.

179. Quartz-Topp J. Implementing quality improvement through knowledge brokering: a Dutch case study. Leadership in Health Services. 2019;32(1):54-68.

180. Reszel J, Dunn SI, Sprague AE, Graham ID, Grimshaw JM, Peterson WE, et al. Use of a maternal newborn audit and feedback system in Ontario: a collective case study. BMJ Qual Saf. 2019;28(8):635-44.

181. Reynolds TL, DeLucia PR, Esquibel KA, Gage T, Wheeler NJ, Randell JA, et al. Evaluating a handheld decision support device in pediatric intensive care settings. JAMIA open. 2019;2(1):49-61.

182. Robertson A, Cresswell K, Takian A, Petrakaki D, Crowe S, Cornford T, et al. Implementation and adoption of nationwide electronic health records in secondary care in England: qualitative analysis of interim results from a prospective national evaluation. Bmj. 2010;341.

183. Robinson TE, Janssen A, Harnett P, Museth KE, Provan PJ, Hills DJ, et al. Embedding continuous quality improvement processes in multidisciplinary teams in cancer care: exploring the boundaries between quality and implementation science. Australian health review. 2016;41(3):291-6.

184. Ruhe MC, Bobiak SN, Litaker D, Carter CA, Wu L, Schroeder C, et al. Appreciative inquiry for quality improvement in primary care practices. Quality management in health care. 2011;20(1):37.

185. Rycroft-Malone J, Seers K, Eldh AC, Cox K, Crichton N, Harvey G, et al. A realist process evaluation within the Facilitating Implementation of Research Evidence (FIRE) cluster randomised controlled international trial: an exemplar. Implementation Science. 2018;13(1):1-15.

186. Saling JC, Atchley DR, Frederick KD, Kiles TM, Rein LJ, Lam HR, et al. Scaling technician product verification: Contextual analysis for developing an implementation strategy for a large community pharmacy chain. Journal of the American Pharmacists Association. 2021;61(5):632-9.

187. Sami S, Amsalu R, Dimiti A, Jackson D, Kenyi S, Meyers J, et al. Understanding health systems to improve community and facility level newborn care among displaced populations in South Sudan: a mixed methods case study. BMC pregnancy and childbirth. 2018;18(1):1-12.

188. Schneider H, English R, Tabana H, Padayachee T, Orgill M. Whole-system change: case study of factors facilitating early implementation of a primary health care reform in a South African province. BMC health services research. 2014;14(1):1-11.

189. Schroeder D, Luig T, Beesoon S, Robert J, Campbell-Scherer D, Brindle M. What work is required to implement and sustain the National Surgical Quality Improvement Program (NSQIP)? A qualitative study of NSQIP implementation in Alberta, Canada. BMJ open. 2021;11(9):e044720.

190. Scott SD, Estabrooks CA, Allen M, Pollock C. A context of uncertainty: how context shapes nurses' research utilization behaviors. Qualitative health research. 2008;18(3):347-57.

191. Sheikh A, Cornford T, Barber N, Avery A, Takian A, Lichtner V, et al. Implementation and adoption of nationwide electronic health records in secondary care in England: final qualitative results from prospective national evaluation in “early adopter” hospitals. Bmj. 2011;343.

192. Shelley KD, Frumence G, Mpembeni R, Mwinnyaa G, Joachim J, Kisusi HK, et al. "Because Even the Person Living With HIV/AIDS Might Need to Make Babies" - Perspectives on the Drivers of Feasibility and Acceptability of an Integrated Community Health Worker Model in Iringa, Tanzania. Int J Health Policy Manag. 2019;8(9):538-49.

193. Shen H, Van Der Kleij RM, Van Der Boog PJ, Wang W, Song X, Li Z, et al. Patients’ and healthcare professionals’ beliefs, perceptions and needs towards chronic kidney disease self-management in China: a qualitative study. BMJ open. 2021;11(3):e044059.

194. Showalter D, Wenger LD, Lambdin BH, Wheeler E, Binswanger I, Kral AH. Bridging institutional logics: Implementing naloxone distribution for people exiting jail in three California counties. Social Science & Medicine. 2021;285:114293.

195. Shrubsole K, Lin TJ, Burton C, Scott J, Finch E. Delivering an iterative Communication Partner Training programme to multidisciplinary healthcare professionals: A pilot implementation study and process evaluation. International Journal of Language & Communication Disorders. 2021;56(3):620-36.

196. Sicotte C, Paré G. Success in health information exchange projects: Solving the implementation puzzle. Social science & medicine. 2010;70(8):1159-65.

197. Simione M, Frost HM, Cournoyer R, Mini FN, Cassidy J, Craddock C, et al. Engaging stakeholders in the adaptation of the connect for health pediatric weight management program for national implementation. Implementation Science Communications. 2020;1:1-13.

198. Sobo E, Bowman C, Halloran J, Aarons G, Asch S, Gifford A. Enhancing organizational change and improvement prospects: Lessons from an HIV testing intervention for veterans. Human Organization. 2008;67(4):443-53.

199. Sopcak N, Aguilar C, O'Brien MA, Nykiforuk C, Aubrey-Bassler K, Cullen R, et al. Implementation of the BETTER 2 program: a qualitative study exploring barriers and facilitators of a novel way to improve chronic disease prevention and screening in primary care. Implement Sci. 2016;11(1):158.

200. Sperber NR, Bruening RA, Choate A, Mahanna E, Wang V, Powell BJ, et al. Implementing a mandated program across a regional health care system: a rapid qualitative assessment to evaluate early implementation strategies. Quality management in health care. 2019;28(3):147.

201. Staras SA, Kastrinos AL, Wollney EN, Desai S, O’Neal LTJ, Johnson-Mallard V, et al. Differences in stakeholder-reported barriers and implementation strategies between counties with high, middle, and low HPV vaccine initiation rates: a mixed methods study. Implementation Science Communications. 2022;3(1):95.

202. Steketee AM, Archibald TG, Harden SM. Adjust your own oxygen mask before helping those around you: an autoethnography of participatory research. Implementation Science. 2020;15:1-20.

203. Stewart EE, Nutting PA, Crabtree BF, Stange KC, Miller WL, Jaén CR. Implementing the patient-centered medical home: observation and description of the National Demonstration Project. The Annals of Family Medicine. 2010;8(Suppl 1):S21-S32.

204. Stime KJ, Garrett N, Sookrajh Y, Dorward J, Dlamini N, Olowolagba A, et al. Clinic flow for STI, HIV, and TB patients in an urban infectious disease clinic offering point-of-care testing services in Durban, South Africa. BMC health services research. 2018;18(1):363.

205. Takian A, Sheikh A, Barber N. Organizational learning in the implementation and adoption of national electronic health records: Case studies of two hospitals participating in the National Programme for Information Technology in England. Health informatics journal. 2014;20(3):199-212.

206. Tarrant C, O’Donnell B, Martin G, Bion J, Hunter A, Rooney KD. A complex endeavour: an ethnographic study of the implementation of the Sepsis Six clinical care bundle. Implementation Science. 2016;11(1):1-11.

207. Taylor F, Galloway S, Irons K, Mess L, Pemberton L, Worton K, et al. Barriers and enablers to implementation of the therapeutic engagement questionnaire in acute mental health inpatient wards in England: A qualitative study. International Journal of Mental Health Nursing. 2022;31(6):1467-79.

208. Thies K, Schiessl A, Khalid N, Hess AM, Harding K, Ward D. Evaluation of a learning collaborative to advance team-based care in Federally Qualified Health Centers. BMJ Open Qual. 2020;9(3).

209. Thompson C, McCaughan D, Cullum N, Sheldon T, Raynor P. Barriers to evidence-based practice in primary care nursing--why viewing decision-making as context is helpful. J Adv Nurs. 2005;52(4):432-44.

210. Thompson-Lastad A, Atreya CE, Chao MT, Pollak C, Dhruva A, Santana T, et al. Improving Access to Integrative Oncology Through Group Medical Visits: A Pilot Implementation Project. J Altern Complement Med. 2019;25(7):733-9.

211. Urquhart R, Porter GA, Sargeant J, Jackson L, Grunfeld E. Multi-level factors influence the implementation and use of complex innovations in cancer care: a multiple case study of synoptic reporting. Implementation Science. 2014;9(1):1-16.

212. Vamos CA, Griner SB, Kirchharr C, Green SM, DeBate R, Daley EM, et al. The development of a theory-based eHealth app prototype to promote oral health during prenatal care visits. Translational behavioral medicine. 2019;9(6):1100-11.

213. Van Tiem JM, Schacht Reisinger H, Friberg JE, Wilson JR, Fitzwater L, Panos RJ, et al. The STS case study: an analysis method for longitudinal qualitative research for implementation science. BMC medical research methodology. 2021;21(1):1-12.

214. Van Twillert S, Postema K, Geertzen JH, Hemminga T, Lettinga AT. Improving rehabilitation treatment in a local setting: a case study of prosthetic rehabilitation. Clinical rehabilitation. 2009;23(10):938-47.

215. Wæhle HV, Haugen AS, Wiig S, Søfteland E, Sevdalis N, Harthug S. How does the WHO Surgical Safety Checklist fit with existing perioperative risk management strategies? An ethnographic study across surgical specialties. BMC health services research. 2020;20(1):1-11.

216. Waitzkin H, Getrich C, Heying S, Rodríguez L, Parmar A, Willging C, et al. Promotoras as mental health practitioners in primary care: a multi-method study of an intervention to address contextual sources of depression. Journal of community health. 2011;36(2):316‐31.

217. Wakerman J, Chalmers EM, Humphreys JS, Clarence CL, Bell AI, Larson A, et al. Sustainable chronic disease management in remote Australia. Med J Aust. 2005;183:S64-8.

218. Waring J, Bishop S, Marshall F, Tyler N, Vickers R. An ethnographic study comparing approaches to inter-professional knowledge sharing and learning in discharge planning and care transitions. Journal of Health Organization and Management. 2019;33(6):677-94.

219. Warren CE, Ndwiga C, Sripad P, Medich M, Njeru A, Maranga A, et al. Sowing the seeds of transformative practice to actualize women's rights to respectful maternity care: reflections from Kenya using the consolidated framework for implementation research. BMC Womens Health. 2017;17(1):69.

220. Whybrow R, Webster L, Girling J, Brown H, Wilson H, Sandall J, et al. Implementation of national antenatal hypertension guidelines: a multicentre multiple methods study. BMJ open. 2020;10(10):e035762.

221. Willms DG, Arratia MI, Makondesa P. Malawi faith communities responding to HIV/AIDS: preliminary findings of a knowledge translation and Participatory-Action Research (PAR) project. Afr J AIDS Res. 2004;3(1):23-32.

222. Wu RC, Lo V, Morra D, Wong BM, Sargeant R, Locke K, et al. The intended and unintended consequences of communication systems on general internal medicine inpatient care delivery: a prospective observational case study of five teaching hospitals. J Am Med Inform Assoc. 2013;20(4):766-77.

223. Wyse JJ, Mackey K, Lovejoy TI, Kansagara D, Tuepker A, Gordon AJ, et al. Expanding access to medications for opioid use disorder through locally-initiated implementation. Addiction Science & Clinical Practice. 2022;17(1):1-11.

224. Yee WL, Htay H, Mohamed Y, Nightingale CE, Tin HH, Thein W, et al. Operational experiences associated with the implementation of near point-of-care early infant diagnosis of HIV in Myanmar: a qualitative study. BMC health services research. 2021;21(1):1-9.

225. Yue J, Liu J, Williams S, Zhang B, Zhao Y, Zhang Q, et al. Barriers and facilitators of kangaroo mother care adoption in five Chinese hospitals: a qualitative study. BMC public health. 2020;20(1):1234.

226. Zakumumpa H, Kwiringira J, Rujumba J, Ssengooba F. Assessing the level of institutionalization of donor-funded anti-retroviral therapy (ART) programs in health facilities in Uganda: implications for program sustainability. Glob Health Action. 2018;11(1):1523302.

227. Zhang K, Chia K, Hawley CE, Uricchio MJ, Driver JA, Salow M. A blueprint for success: using an implementation framework to create a medication history technician pilot program. Journal of the American Pharmacists Association. 2021;61(4):e301-e15.
